# Identifying critical state of complex diseases by single-sample Kullback–Leibler divergence

**DOI:** 10.1186/s12864-020-6490-7

**Published:** 2020-01-28

**Authors:** Jiayuan Zhong, Rui Liu, Pei Chen

**Affiliations:** School of Mathematics, South China University of Technology, Guangzhou, 510640 China

**Keywords:** Tipping point, Dynamic network biomarker (DNB), Pre-disease state, Critical transition, Single-sample Kullback–Leibler divergence (sKLD)

## Abstract

**Background:**

Developing effective strategies for signaling the pre-disease state of complex diseases, a state with high susceptibility before the disease onset or deterioration, is urgently needed because such state usually followed by a catastrophic transition into a worse stage of disease. However, it is a challenging task to identify such pre-disease state or tipping point in clinics, where only one single sample is available and thus results in the failure of most statistic approaches.

**Methods:**

In this study, we presented a single-sample-based computational method to detect the early-warning signal of critical transition during the progression of complex diseases. Specifically, given a set of reference samples which were regarded as background, a novel index called single-sample Kullback–Leibler divergence (sKLD), was proposed to explore and quantify the disturbance on the background caused by a case sample. The pre-disease state is then signaled by the significant change of sKLD.

**Results:**

The novel algorithm was developed and applied to both numerical simulation and real datasets, including lung squamous cell carcinoma, lung adenocarcinoma, stomach adenocarcinoma, thyroid carcinoma, colon adenocarcinoma, and acute lung injury. The successful identification of pre-disease states and the corresponding dynamical network biomarkers for all six datasets validated the effectiveness and accuracy of our method.

**Conclusions:**

The proposed method effectively explores and quantifies the disturbance on the background caused by a case sample, and thus characterizes the criticality of a biological system. Our method not only identifies the critical state or tipping point at a single sample level, but also provides the sKLD-signaling markers for further practical application. It is therefore of great potential in personalized pre-disease diagnosis.

## Background

**Fig. 1:**
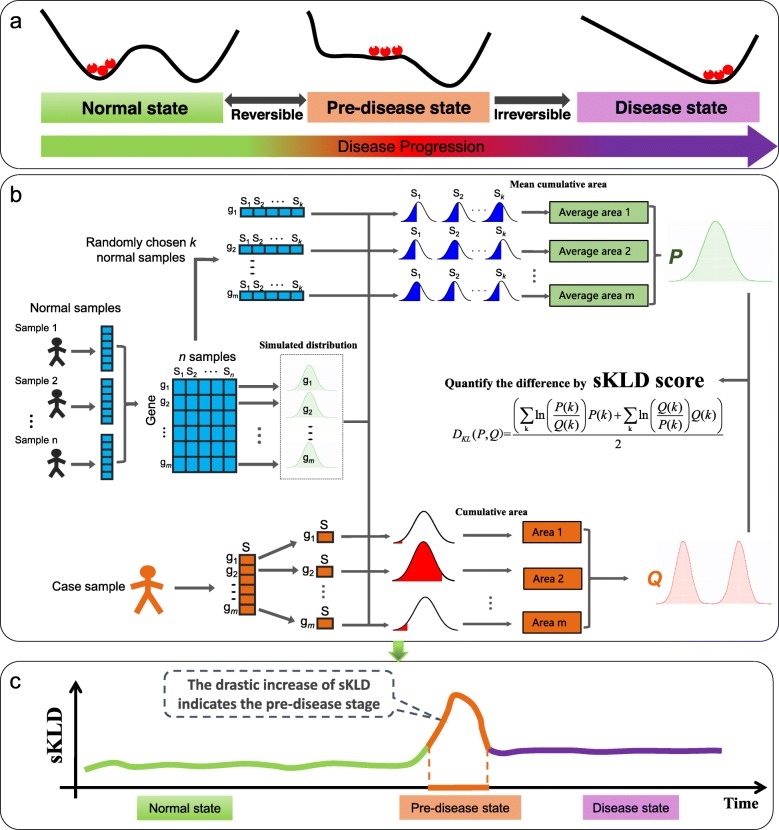
The outline for detecting the early-warning signal of a pre-disease state based on sKLD. **a** The progression of complex diseases is modeled as three states, including two stable states, i.e., a normal and a disease state with high stability and resilience, and an unstable pre-disease state with low stability and resilience [5, 9]. As the limit of the normal state, the pre-disease state is a critical state just before the onset of deterioration. **b** Given a number of reference samples that are generally from normal cohort and represent the healthy or relatively healthy individuals, the sKLD score is capable to quantitatively evaluate the difference between two distributions of each gene, i.e., the background distribution that generated from a set of reference samples, and a perturbed distribution yielded from the single case sample. The detailed procedure and description of deriving the two distributions are presented in Methods section. **c** During the progression of a complex disease, the pre-disease state is indicated by the significant change of sKLD, i.e., the sKLD changes gradually when the system is in the normal state, while it increases abruptly when the system approaches the tipping point

Critical transitions are sudden and large-scale state transitions that occur in many complex systems, such as ecological systems [1, 2], climate systems [3, 4], financial markets [5, 6], microorganism populations [7], psychiatric conditions [8],infectious disease spreading [9] and the human body [10]. Recently, considerable evidence suggests that during the progression of many complex diseases, e.g. cancer [11], asthma attacks [12], epileptic seizures [13] the deterioration is not always smooth but abrupt, inferring the existence of a so-called tipping point, at which a drastic or qualitative transition may occur. Accordingly, the progression of a complex disease can be roughly divided into three stages regardless of specific biological and pathological differences during the progression of diseases, that is, (1) a normal state, a steady state representing the relatively healthy stage and with high resilience; (2) a pre-disease state, which is the limit of the normal state immediately before the onset of deterioration, and with low resilience and high susceptibility; and (3) a disease state, the other steady state with high resilience after the qualitative deterioration (Fig. 1a). It is important to predict the tipping point, so as to prevent or at least get ready for the upcoming deterioration by taking appropriate intervention actions. Recently, we proposed a theoretic framework, called the dynamical network biomarker (DNB) concept [10, 14] for identifying the pre-disease state of complex diseases. This DNB concept, directly from the critical slowing-down theory [15, 16], provides statistical method to select relevant variables for the pre-disease state, that is, a small group of closely related variables (DNBs) convey early warning signals for the impending critical transition by some drastic statistical indices [17, 18]. The DNB theory and its extensions have been applied to several cases, detected the tipping points of endocrine resistance [19] as well as cellular differentiation [20], investigated the immune checkpoint blockade [21], and helped to find the corresponding pre-disease states of several diseases [18, 22–26]. However, DNB method requires multiple samples at each time point, which are generally not available in clinics and other practical cases, thus significantly restricting the application of DNB method in most real cases. Therefore, when there is only a single case sample available, it requires new computational method to explore the critical information, detect the early-warning signal and identify the pre-disease state.

The rapid development of high-throughput technology provides new insights for computational analysis, even when there is only one single sample available. Actually, based on a sample of high-throughput data, it is possible to measure the expressions of thousands of genes simultaneously. Such high-dimensional observation at the genome-wide scale not only provides the global view of a biological system, but also presents the accumulated effects of its long-term dynamics. Motivated by this point, in this study we develop a data-driven computational method and achieve the single-sample detection of the pre-disease state, by exploring the rich dynamical information from the high-throughput omics data. Specifically, it is found that the qualitative state change often causes the significant changes in the distributions of some genes’ expression. Therefore, a novel index, the single-sample Kullback–Leibler divergence (sKLD), is proposed to quantify the disturbance brought by the single case sample on the background distribution, where the background or reference samples refer to samples collected from a few healthy/relatively healthy individuals. Correspondingly, an applicable algorithm is developed based on sKLD (Fig. 1b), including a procedure of simulating the background distribution for each gene, evaluating the perturbation to the background distribution triggered by a single case sample, detecting the early-warning signal and identifying the pre-disease state. During this procedure, a group of biomolecules whose expressions are highly fluctuating before the critical transition are also picked out as the sKLD-signaling marker for further practical application. This new approach has been applied to a numerical simulation, and six real datasets including lung squamous cell carcinoma (LUSC), lung adenocarcinoma (LUAD), stomach adenocarcinoma (STAD), thyroid carcinoma (THCA), colon adenocarcinoma (COAD) from the cancer genome atlas (TCGA) database and acute lung injury (GSE2565) from the NCBI GEO database. The identified pre-disease states all agree with the experimental observation or survival analysis. And the corresponding signaling markers have been validated by functional enrichment.

## Results

We present the definition and algorithm of sKLD score in Methods section. Here, we used a single-sample with high-throughput omics data, to identify the pre-disease state or early warning signals of the disease deterioration based on the sKLD score. Achieving reliable identification with only one sample is of great importance in clinic application since it is usually difficult to obtain multiple samples from an individual who does not yet exhibit any disease symptoms during a short period. To illustrate how sKLD works, we applied our method first to a simulated dataset, and then to six real datasets, including LUSC, LUAD, STAD, THCA and COAD from TCGA database (http://cancergenome.nih.gov) and acute lung injury (GSE2565) from the GEO database (http://www.ncbi.nlm.nih.gov/geo/). The successful identification of the pre-disease states in these diseases validated the effectiveness of sKLD method in quantifying the tipping point just before the critical transitions into severe disease states.

### Validation based on numerical simulation

**Fig. 2:**
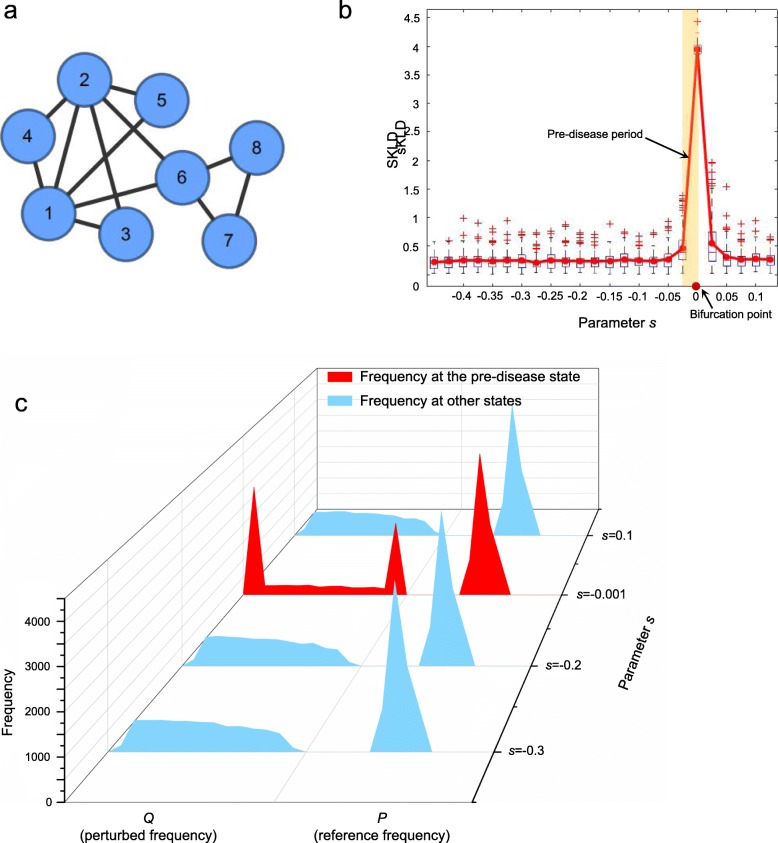
The performance of sKLD based on a dataset of numerical simulation. **a** A network with eight nodes governed by a model is represented in Michaelis-Menten form, based on which the numerical simulation is conducted. **b** The curve of sKLD score defined in Eq. (2). It is obvious that the sKLD would abruptly increase when the system is near the critical point, i.e., *s* = 0, which is in accordance with the bifurcation parameter value at *s* = 0 (see Eq. (S3) in Additional file 1: A). **c** It is seen that the perturbed frequency *Q* presents two peaks when the system approaches the tipping point, i.e., *s* = 0, comparing with that in a normal state (*s* = − 0.2) or a disease state (*s* = 0.1) and there is no significant difference in three stages of disease progression for the reference *P*

A model of an eight-node artificial network (Fig. 2a) was used to validate the proposed computational method. This network is the regulatory representation for a set of eight biomolecules, governed by eight stochastic differential equations Eq. (S1) shown in Additional file 1: A. Such a model is represented in Michaelis-Menten form. This type of regulatory network is usually applied to study genetic regulations including transcription and translation processes [27–29], and multi-stability and nonlinear biological processes [30, 31]. In addition, the bifurcation in Michaelis-Menten form is often employed to model the state transition of gene regulatory networks [32, 33]. In Eq. (S1), a parameter *s* was varying from − 0.5 to 0.2. Based on this model, a numerical simulation dataset was generated.

It is seen in Fig. 2b that the single-sample Kullback–Leibler divergence (sKLD) abrupt increases when the system approaches a special parametric value *s* = 0, which was set as a Hopf bifurcation value (see Additional file 1: A for details). In other word, the high level of sKLD in the vicinity of the critical parameter value *s* = 0 represents that the reference distribution *P* is significantly different from the perturbed distribution *Q*, which was generated from a single pre-disease sample. Besides, to demonstrate the robustness of the proposed method, a hundred sKLD scores were calculated for each parameter *s*, respectively based on a hundred single samples perturbed by additive white noise. It is seen that the median values of the box plots in Fig. 2b also stably provides signals for the tipping point, which indicates the sKLD score is featured with robustness against sample noises. To better illustrate the different distribution between normal and pre-disease states, the dynamical progression of frequencies *P* and *Q* were demonstrated in Fig. 2c with a series of parametric values, i.e., *s* ∈ {−0.3, −0.2, −0.001, 0.1}. Each frequency in Fig. 2c is a statistical plot based on ten thousand simulations. From these frequency plots, it suggests that the perturbed frequency *Q* in a pre-disease state (*s* = 0) presents two peaks, that is, when the network system is in a pre-disease state, the expressions of some nodes wildly fluctuate in a strongly collective manner, resulting a distinct distribution. This critical phenomenon is accurately detected by sKLD, which quantitatively provides a score for identifying the upcoming bifurcation point. Therefore, the numerical simulation validated the effectiveness of sKLD in detecting the early warning signal of a qualitative state transition. The detailed dynamical system is proposed in Additional file 1: A. The source code of numerical simulation is accessed in https://github.com/zhongjiayuna/KL_Project.

### Identifying the critical transition for acute lung injury

**Fig. 3:**
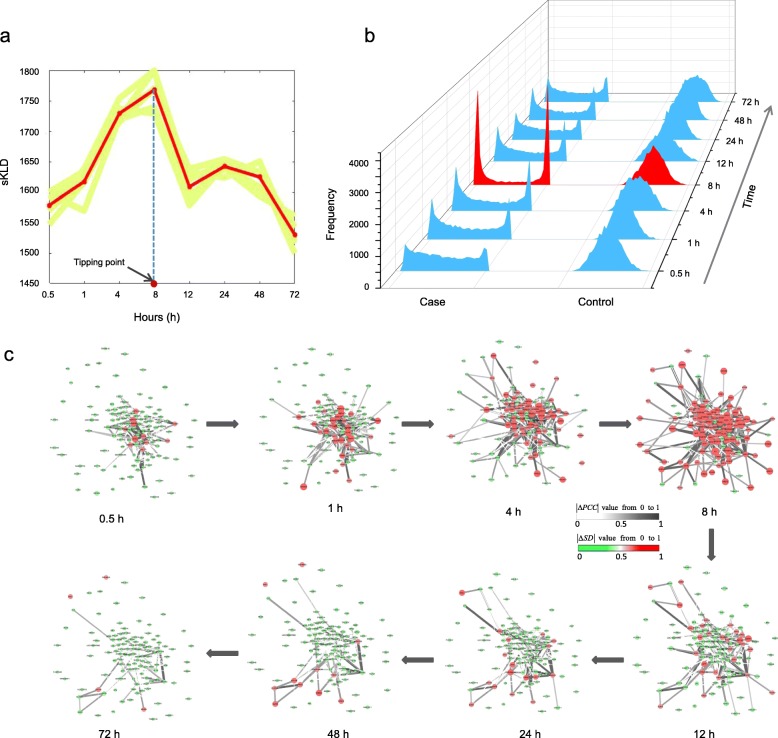
The application of sKLD in acute lung injury. **a** As shown in the red curve, the peak for the sKLD appears at 8 h, which can be used as an early signal of acute lung injury deterioration. The result is consistent with the experimental observation. To illustrate the significance of the result, six yellow curves are derived based on six sets of datasets generated from a leave-one-out scheme, which consistently indicate the tipping point at 8 h. **b** The figure shows the dynamical changes in the distribution of signaling genes for the case data and control data, respectively. **c** From the dynamical evolution of the network composed by the top 5% most significant genes in terms of the cumulative area of the case sample, it is seen that the an obvious change in the network structure appear at 8 h

The sKLD has been applied to the microarray data of dataset GSE2565, which is obtained from a mouse experiment of phosgene-induced acute lung injury [34]. In the original experiment, the gene expression data of case samples were derived from the lung tissues of CD-1 male mice exposed to phosgene up to 72 h, while the data of control samples were from that exposed to air. During the experiment for both case and control groups, there are totally nine sampling points, i.e., 0, 0.5, 1, 4, 8, 12, 24, 48, and 72 h, while at each sampling time point, lung tissues were obtained from six mice [34]. Applying the proposed sKLD-based method to the dataset, we regard the six samples at the first time point (0 h) as the reference/normal samples for both case and control groups. The mean sKLD score shown as the red curve in Fig. 3a, abruptly increases and reaches a peak at 8 h, suggesting that there is a critical transition around 8 h. To demonstrate the significance of the result, six datasets were generated from a leave-one-out scheme. Applying the sKLD algorithm to these datasets respectively, six mean sKLD scores were derived and plotted as the yellow curves in Fig. 3a. It is seen that these sKLD curves based on the re-sampled datasets all indicates the tipping point at 8 h. In Fig. 3b, it exhibits the dynamical change of distributions for both case and control samples. Obviously for control samples, there is little dynamical difference in the perturbed distributions, while for case samples, the perturbed distribution at the 4th sampling time point (8 h) is notably distinct from that at other sampling time points (Fig. 3b), leading to the significant change of sKLD score of case samples at 8 h. The abrupt change of such quantitative index demonstrates its effectiveness in detecting early signals of critical transition for complex diseases at a network level, which may also reveal the mechanisms on disease progression [35–37]. In Fig. 3c, we demonstrate the dynamical evolution of a network composed by the top 5% most significant genes in terms of the cumulative area of the case sample. Clearly, an obvious change in the network structure occurs around 8 h, signaling the upcoming critical transition at the network level. These results agree with the observation in original experiment, that is, after 8-h exposure to phosgene, the mice in case group were observed a series of symptoms including enhanced bronchoalveolar lavage fluid (BALF) protein levels, increased pulmonary edema, and ultimately decreased survival rates [34]. The severe phosgene-induced acute lung injury is around 8 h and lasts until 12 h after exposure. About 50–60% deaths were observed after 12-hous exposure, and 60–70% mortality was observed after 24-h exposure [34]. Comparing with the former DNB method [10], the common signaling genes for acute lung injury is provided in Additional file 3.

### Identifying the critical transition for tumor diseases

To demonstrate the effectiveness, the proposed sKLD method is applied to five tumor datasets, lung squamous cell carcinoma (LUSC), lung adenocarcinoma (LUAD), stomach adenocarcinoma (STAD), thyroid carcinoma (THCA), colon adenocarcinoma (COAD) from the cancer genome atlas (TCGA), all of which were composed by tumor and tumor-adjacent samples. The tumor samples were grouped into different cancer stages according to corresponding clinical information of TCGA, that is, the tumor samples were classified into seven stages for LUSC, LUAD and STAD, and four stages for THCA and COAD. The detailed sampling conditions are provided in Additional file 1: Table S1. In all the five datasets, the tumor-adjacent samples were employed as normal/reference samples. The sKLD was then calculated for each single tumor sample following the proposed algorithm (the five steps) in Methods. Finally, the average sKLD of each stage was taken to identify any possible critical/pre-transition state.

**Fig. 4:**
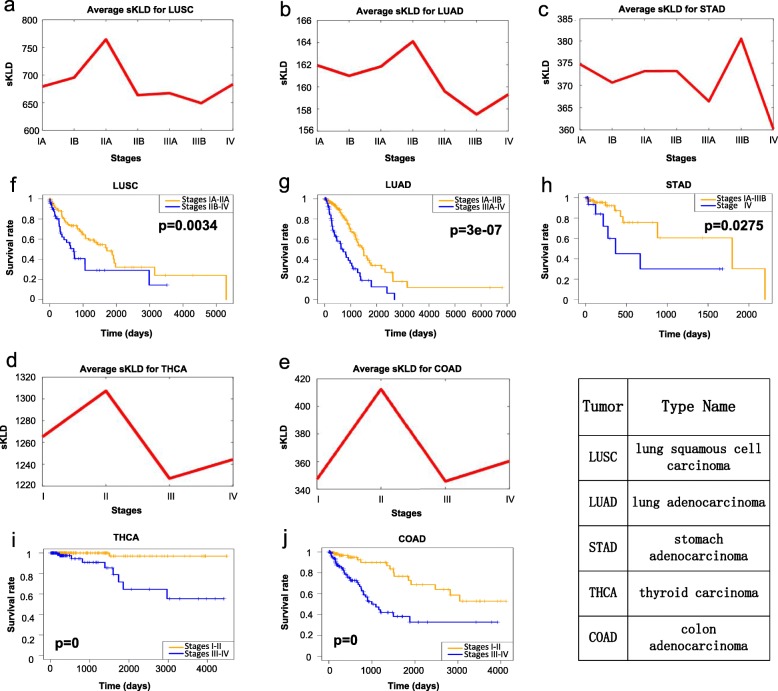
Identification of critical transition for tumor deterioration in five cancers: **a** LUSC, **b** LUAD, **c** STAD, **d** THCA and **e** COAD. Comparison of survival curves before and after critical state for five cancers: **f** LUSC, **g** LUAD, **h** STAD, **i** THCA and **j** COAD

Clearly, the significant change of sKLD successfully indicated the critical stages prior to the metastasis for all the five cancers (Fig. 4a-e). To validate the identified critical state, the prognosis results respectively based on before-transition and after-transition samples were exhibited and compared through Kaplan-Meier (log-rank) survival analysis (Fig. 4f-j and Additional file 1: Figure S4). Specifically, before the identified critical stage, there is generally a high expectation of life after diagnosis, while after the critical stage, there is a much lower expectation of survival after diagnosis (Fig. 4f-j). However, before and after any other stages, there was no significant difference in the prognosis (Additional file 1: Figure S4), which suggests that the identified critical stage is accurate and closely associated with prognosis.

### The critical state of LUSC

For LUSC, the sKLD score abruptly increases at stage IIA (Fig. 4a), indicating an upcoming critical transition after stage IIA, that is, the invasion into the mediastinal pleura at stage IIB, after which there are lymph nodes metastasis, tumor invaded the visceral pericardial surface and the intrapericardial pulmonary artery [38]. The critical transition has also been validated by survival analysis. It is seen from Fig. 4f that the survival time of before-transition samples (samples from stages IA-IIA) is much longer than that of after-transition samples (samples from stages IIB-IV), resulting significant difference (significant value *p* = 0.0034) between the survival curves of two sets of samples, i.e., samples derived before and after stage IIA of LUSC. For the samples solely from the two stages around the critical transition point, i.e., stages IIA and IIB, the survival time of stage-IIA samples is longer than that of stage-IIB samples (*p* = 0.036; Additional file 1: Figure S5a). Besides, to check if there is any other critical transition that leads to different survival time, a series of survival analysis has been carried out. As shown in Additional file 1: Figure S5b-S5c, statistically there is little difference (*p* = 0.4741; Additional file 1: Figure S5b) between the survival time of stages-IA samples and that of stage-IB samples, and little statistical differences (*p* = 0.5671; Additional file 1: Figure S5c) in survival time among samples from stages IIB, IIIA, IIIB, IV. In other word, there is no other critical transition point in either before-transition period (stages IA-IB), or after-transition period (stages IIB-IV). These results demonstrate that given high-throughput molecular data, the critical transition associated with disease deterioration and survival time in LUSC can be identified by sKLD.

In addition, at the identified critical stage (stage IIA), the top 5% most significant genes in terms of the cumulative area of the case sample are selected as “sKLD-signaling genes” for further functional analysis. Some genes in the common “sKLD-signaling genes” have been reported to be associated with the process of LUSC (Table 1). For instance, the miR-195 axis regulates lung squamous cell carcinoma (LUSC) progression through BIRC5 [39]. CCNA2 promotes invasion and migration of non-small cell lung cancer cells through integrin αvβ3 signaling pathway [40]. miR-26a/b inhibits directly migration, invasion, and proliferation of lung cancer cells by targeting CDC6 [41]. CKS1B is a lung cancer-related gene, knockdown of which results in a significant decrease in lung cancer cell proliferation, invasion and migration [42]. Depletion of E2F8 inhibits cell proliferation and tumor growth in lung cancer, thus E2F8 can be considered as a novel therapeutic target for lung cancer [43]. Knockdown of FOXM1 inhibits the cell proliferation in LUSC [44]. ITPKA serves as an early diagnostic marker in lung cancer, whose overexpression promotes tumorigenesis [45]. MCM2 regulates proliferation and cell cycle in lung squamous cell carcinoma, whose overexpressed protein is obviously associated with malign differentiated degree and lymph node metastasis [46]. The sKLD-signaling genes for the five tumor datasets were provided in Additional file 2.

**Table 1:** The genes with high frequency in 13 “sKLD-signaling genes” groups in the critical stage (stage IIA) for LUSC

Gene	Frequency	Location	Family*	Relation with cancer progression
BIRC5	13	Cytoplasm	other	The miR-195 axis regulates lung squamous cell carcinoma (LUSC) progression through BIRC [39].
CCNA2	13	Nucleus	other	CCNA2 promotes invasion and migration of non-small cell lung cancer cells through integrin αvβ3 signaling pathway [40].
CDC6	13	Nucleus	other	miR-26a/b inhibits directly migration, invasion, and proliferation of lung cancer cells by targeting CDC6 [41].
CKS1B	13	Other	kinase	CKS1B is a lung cancer-related gene, knockdown of which can result in a significant decrease in lung cancer cell proliferation, invasion and migration [42].
E2F8	13	Nucleus	transcription regulator	Depletion of E2F8 inhibits cell proliferation and tumor growth in lung cancer, thus E2F8 can be considered as a novel therapeutic target for lung cancer [43].
FOXM1	13	Nucleus	transcription regulator	Knockdown of FOXM1 inhibits the cell proliferation in LUSC [44].
ITPKA	13	Cytoplasm	kinase	ITPKA serves as an early diagnostic marker in lung cancer, whose overexpression promotes tumorigenesis [45].
MCM2	13	Nucleus	enzyme	MCM2 regulates proliferation and cell cycle in lung squamous cell carcinoma, whose overexpressed protein is obviously associated with malign differentiated degree and lymph node metastasis [46].

Besides, functional enrichment through GO analysis shows that the common sKLD-signaling genes are involved in the biological processes including cytoskeleton organization, chromosome condensation, regulation of cell division and others (Table 2). These biological processes are associated with the progression of cancer. Furthermore, through IPA (Ingenuity Pathway Analysis), these common genes are also enriched to cancer-related function annotation, such as lung squamous cell carcinoma, development of malignant tumor and lung carcinoma (Table 2).

**Table 2:** The functional enrichment of common “sKLD-signaling genes” in the critical stage samples for LUSC

Gene Ontology Consortium		IPA	
enriched biological process	enriched *p* value	enriched biological process	enriched *p* value
cytoskeleton organization (GO:0007010)	2.00E-12	mitosis of tumor cell lines	1.39E-39
chromosome condensation (GO:0030261)	4.49E-09	development of malignant tumor	6.37E-29
regulation of cell division (GO:0051302)	1.23E-08	lung squamous cell carcinoma	1.62E-24
sister chromatid cohesion (GO:0007062)	2.43E-06	lung carcinoma	7.28E-22
isotype switching (GO:0045190)	1.03E-04	respiratory system tumor	1.19E-19
programmed cell death (GO:0012501)	6.57E-04	lymphocytic neoplasm	9.89E-12

### The critical state of LUAD

In Fig. 4b, the peak of the sKLD score at stage IIB suggests that there is a critical transition of LUAD after stage IIB. References showed that after stage IIB, ipsilateral mediastinal or subcarinal lymph nodes were metastasized (stage IIIA) and tumor began to invade heart, great vessels and trachea (stages IIIA-IIIB) [47]. As shown in Fig. 4g, there is significant difference (*p* = 3E-07) between the survival curves of samples before and after stage IIB of LUAD. Clearly, the survival time of before-transition samples (samples from stages IA-IIB) is considerably longer than that of after-transition samples (samples from stages IIIA-IV). For the samples solely from two stages IIB and IIIA around the critical transition point, the survival time of stage-IIB samples is much longer than stage-IIIA samples (*p* = 0.012; Additional file 1: Figure S5d). Besides, statistically it shows little significant difference (*p* = 0.4421; Additional file 1: Figure S5e) among the survival curves of samples from stages IA, IB, IIA (the stages before the critical state), and little difference (*p* = 0.1649; Additional file 1: Figure S5f) among the survival curves of samples from stages IIIA, IIIB, IV (the stages after the critical state), which show that stage IIB of LUAD is highly associated with the critical transition of survival time.

The functional analysis is carried out based on “sKLD-signaling genes”. Through literature searching, some genes in the common “sKLD-signaling genes” have been shown to be associated with the process of LUAD (Table 3). For example, PYCR1 may be a novel therapeutic target for inhibiting cell proliferation in lung cancer [48]. ETV4 promotes proliferation and invasion of lung adenocarcinoma by transcriptionally upregulating MSI2 [49]. Knockdown of PITX2 inhibits cell proliferation, migration and invasion of LUAD [50]. MDK plays an important role in non-small cell lung cancer progression and prognosis and may act as a convincing prognostic indicator for non-small cell lung cancer patients [51]. Blocking glutamine-mediated induction of PPAT inhibits cell proliferation and invasion in LUAD [52]. TOP2A is an ideal candidate as miR-144-3p target in non-small cell lung cancer, while MiR-144-3p expression is significantly correlated with lymph node metastasis and vascular invasion [53]. HOXC13 promotes proliferation of lung adenocarcinoma via modulation of CCND1 and CCNE1 [54]. Up-regulation of SRPK1 in non-small cell lung cancer promotes the growth and migration of cancer cells [55].

**Table 3:** The genes with high frequency in 59 “sKLD-signaling genes” groups in the critical stage (stage IIB) for LUAD

Gene	Frequency	Location	Family*	Relation with cancer progression
PYCR1	54	Cytoplasm	enzyme	PYCR1 may be a novel therapeutic target for inhibiting cell proliferation in lung cancer [48].
ETV4	50	Nucleus	transcription regulator	ETV4 promotes proliferation and invasion of lung adenocarcinoma by transcriptionally upregulating MSI2 [49].
PITX2	50	Nucleus	transcription regulator	Knockdown of PITX2 inhibits cell proliferation, migration and invasion of LUAD [50].
MDK	49	Extracellular Space	growth factor	MDK plays an important role in non-small cell lung cancer progression and prognosis and may act as a convincing prognostic indicator for non-small cell lung cancer patients [51].
PPAT	49	Cytoplasm	enzyme	Blocking glutamine-mediated induction of PPAT inhibits cell proliferation and invasion in LUAD [52].
TOP2A	49	Nucleus	enzyme	TOP2A is an ideal candidate for miR-144-3p targets in non-small cell lung cancer and MiR-144-3p expression is significantly correlated with stage, lymph node metastasis and vascular invasion [53].
HOXC13	48	Nucleus	transcription regulator	HOXC13 promotes proliferation of lung adenocarcinoma via modulation of CCND1 and CCNE1 [54].
SRPK1	48	Nucleus	kinase	Up-regulation of SRPK1 in non-small cell lung cancer can promote the growth and migration of cancer cells [55].

Moreover, functional enrichment through GO analysis shows that the common “sKLD-signaling genes” are involved in the biological processes of meiotic cell cycle, cell cycle checkpoint, cytokinesis, and so on (Table 4). These biological processes are associated with the progression of cancer. In addition, these common genes were also related to lung adenocarcinoma and development of lung tumor by functional enrichment in IPA (Table 4).

**Table 4:** The functional enrichment of common “sKLD-signaling genes” in the critical stage samples for LUAD

Gene Ontology Consortium		IPA	
enriched biological process	enriched *p* value	enriched biological process	enriched *p* value
cell cycle checkpoint (GO:0000075)	1.92E-15	Lung cancer	7.77E-28
meiotic cell cycle (GO:0051321)	1.12E-11	Lung tumor	2.97E-27
cytokinesis (GO:0000910)	1.71E-06	Lung carcinoma	1.42E-23
fucosylation (GO:0036065)	1.65E-04	Non-small cell lung carcinoma	6.74E-22
regulation of cell development (GO:0060284)	3.15E-04	Development of lung tumor	1.48E-21
regulation of developmental process (GO:0050793)	9.30E-04	Lung adenocarcinoma	1.85E-21

### The critical state of STAD

For STAD, as shown in Fig. 4c, the drastic transitions of average sKLD appeared in stage IIIB, which indicated the imminent critical transition at stage IV. According to the division of clinical stages of STAD, the deterioration into stage IV means an advanced metastatic stage, in which the tumor has spread to nearby tissues or metastasized to other parts of the human body [56]. Fig. 4h shows that there is significantly difference (*p* = 0.0257) between the survival time of two group of samples, i.e., samples respectively from the before-transition period (stages IA-IIIB) and from the after-transition period (stages IV) of STAD. It is also noted that the survival time of samples from stage IIIB is significantly longer than that from stage IV (*p* = 0.0215; Additional file 1: Figure S5 g). Besides, there is little significant difference (*p* = 0.1252; Additional file 1: Figure S5 h) among survival curves of samples from the period prior to the critical transition, i.e., stages IA-IIIA. These results demonstrate that the sKLD detected the early-warning signals of a critical transition of survive time and distant metastasis at stage IV.

Some “sKLD-signaling genes” have been found in literatures and identified to be associated with the process of STAD (Table 5). For instance, COL10A1 promotes invasion and metastasis in gastric cancer through transcriptional regulation of SOX9 and the involvement of the TGF-β signaling pathway [57]. BGN promotes tumor invasion and metastasis of gastric cancer both in vitro and in vivo [58]. CTHRC1 may be associated with metastasis in human gastric cancer [59]. Let-7b inhibits cell proliferation, migration, and invasion through targeting CTHRC1 in gastric cancer [60]. Enforced expression of SALL4 not only enhances the proliferation and migration of human gastric cancer cells, but promotes the growth and metastasis of gastric xenograft tumor in vivo [61]. Knockdown of MMP11 inhibits proliferation and invasion of gastric cancer cells [62]. The overexpression of MAP4K4 promotes cancer progression or metastasis [63, 64]. MiR-211 inhibits cell proliferation and invasion of gastric cancer by down-regulating SOX4 [65]. The overexpression of FOXS1 in gastric cancer cell lines can inhibit proliferation, metastasis and epithelial-mesenchymal transition of tumor through downregulating wnt/β-catenin pathway [66].

**Table 5:** The genes with high frequency in 20 “sKLD-signaling genes” groups in the critical stage (stage IIIB) for STAD

Gene	Frequency	Location	Family*	Relation with cancer progression
COL10A1	19	Extracellular Space	other	COL10A1 promotes invasion and metastasis in gastric cancer through transcriptional regulation of SOX9 and the involvement of the TGF-β signaling pathway [57].
BGN	18	Extracellular Space	other	BGN promote tumor invasion and metastasis of gastric cancer both in vitro and in vivo [58].
CTHRC1	18	Extracellular Space	other	CTHRC1 may be associated with metastasis in human gastric cancer [59]. Let-7b inhibits cell proliferation, migration, and invasion through targeting CTHRC1 in gastric cancer [60].
SALL4	18	Nucleus	transcription regulator	Enforced expression of SALL4 not only enhances the proliferation and migration of human gastric cancer cells, but promotes the growth and metastasis of gastric xenograft tumor in vivo [61].
MMP11	18	Extracellular Space	peptidase	Knockdown of MMP11 inhibits proliferation and invasion of gastric cancer cells [62].
MAP4K4	18	Cytoplasm	kinase	MAP4K4 overexpression promotes cancer progression or metastasis [63, 64].
SOX4	18	Nucleus	transcription regulator	MiR-211 inhibits cell proliferation and invasion of gastric cancer by down-regulating SOX4 [65].
FOXS1	17	Nucleus	transcription regulator	Overexpression of FOXS1 in gastric cancer cell lines can inhibit proliferation, metastasis, and epithelial-mesenchymal transition of tumor through downregulating wnt/β-catenin pathway [66].

Besides, based on GO analysis, the common “sKLD-signaling genes” are enriched into the biological processes associated with the progression of cancer, e.g., extracellular matrix organization, collagen fibril organization and ribosome biogenesis (Table 6). Furthermore, according to IPA, the common “sKLD-signaling genes” are also enriched to cancer-related function annotation including digestive organ tumor, digestive system cancer and abdominal cancer (Table 6). The common sKLD-signaling genes for LUSC, LUAD and  STAD were provided in Additional file 4.

**Table 6:** The functional enrichment of common “sKLD-signaling genes” in the critical stage samples for STAD

Gene Ontology Consortium		IPA	
enriched biological process	enriched *p* value	enriched biological process	enriched *p* value
extracellular matrix organization (GO:0030198)	3.32E-14	digestive organ tumor	4.04E-51
collagen fibril organization (GO:0030199)	5.31E-13	digestive system cancer	1.63E-50
ribosome biogenesis (GO:0042254)	1.08E-09	abdominal cancer	2.07E-49
regulation of cell cycle (GO:0051726)	1.61E-08	gastrointestinal tract cancer	7.11E-45
collagen metabolic process (GO:0032963)	3.67E-05	gastrointestinal carcinoma	1.49E-44
regulation of protein ubiquitination (GO:0031396)	9.60E-05	development of digestive organ tumor	5.9E-27

### The critical state of THCA

As shown in Fig. 4d, for THCA, the sKLD score reaches its peak at stage II, signaling the imminent critical transition at stage III. There was extension to sternothyroid muscle or perithyroid soft tissues and regional lymph node metastasis in stage III [67]. There is significant difference between the survival curves before and after stage II in THCA samples (*p* = 0) (Fig. 4i). It is seen that the survival times of samples before the critical state were significantly longer than for samples after the critical state. There was no significant difference in survival curves among samples in stages III, IV (the stages after the critical state) (*p* = 0.5158; Additional file 1: Figure S5i). The survival times of samples in stage II were significantly longer than for samples in stage III (*p* = 0.0381; Additional file 1: Figure S5j). These results illustrate the sKLD can detect the early-warning signals associated with disease deterioration in THCA. Furthermore, the functional analyses of some “sKLD-signaling genes” are performed through IPA and literature searching, which is provided in Additional file 1: Table S2. The enrichment analysis for the common “sKLD-signaling genes” is carried out based on GO and IPA analysis, which is given in Additional file 1: Table S3.

### The critical state of COAD

For COAD, the drastic increase of the sKLD score from stage I to stage II suggests a critical deterioration after stage II (Fig. 4e). There are lymph nodes metastasis and tumor directly invade other organs or structures in stage III [68]. There was significant difference between the survival curves before and after stage II in COAD samples (*p* = 0) (Fig. 4j). As shown in Fig. 4j, the survival time of samples before the critical state were obviously longer than that of samples after the critical state. There were no statistics difference in survival curves among samples in stages III, IV (the stages after the critical state) (*p* = 0.1048; Additional file 1: Figure S5k). The survival times of samples in stage II were significantly longer than for samples in stage III (*p* = 0.0067; Additional file 1: Figure S5 l). These results demonstrate the sKLD can provide the early-warning signals associated with disease deterioration in COAD. Moreover, functional analyses of some “sKLD-signaling genes” are performed through IPA and literature searching, which is given in Additional file 1: Table S4. The enrichment analysis for the common “sKLD-signaling genes” is performed through GO and IPA analysis, which is provided in Additional file 1: Table S5.

## Discussion

Detecting the early-warning signal for the sudden deterioration is crucial to most complex diseases. However, it is a challenging task to identify the pre-disease state prior to the occurrence of obvious symptoms due to the lack of samples, that is, there is usually only one single sample for an individual at a time point before an accurate diagnosis is made. Clearly, such single-sample problem, rising from clinical and experimental practice, leads to the failure of traditional statistic method, and thus requires new approaches that help to overcome the sample limitation. In this study, we proposed a single-sample-based computational framework, the sKLD method, to quantify the disturbance on the background caused by a sample. The sKLD has been applied to real-world datasets and successfully identifies the tipping points or critical states of complex diseases. Specifically, the significant change of sKLD score indicates the pre-disease state of phosgene-induced acute lung injury before the deterioration into pulmonary edema, the critical stage of (stage IIA) of LUSC prior to the lymph nodes metastasis, the critical stage (stage IIB) of LUAD before lymph nodes were metastasized, the critical stage (stage IIIB) of STAD before distant metastasis, the critical stage (stage II) of THCA before lymph node metastasis, and the critical stage (stage II) of THCA before lymph node metastasis. All these identified critical stages were validated by the survival analysis, that is, the patient would have a significantly better prognosis if they were diagnosed before the critical stage. Besides, at any other stages, there was no significant difference in the prognosis, suggesting that the identified critical stage is accurate and closely associated with prognosis. The functional analysis of sKLD-signaling genes is consistent with the upcoming deteriorations of diseases.

There are three advantages of the proposed method. First, in contrast to the traditional biomarkers that are used to “diagnose disease” based on the information of differential expressions, sKLD is capable to “predict disease” based on the information of differential distributions among biomolecules. Second, given some reference samples, sKLD works with only a single sample. Third, it should be noted that sKLD is a model-free method, which implies that in the sKLD strategy there is neither feature selection nor model/parameter training procedure. It is thus different from the traditional machine learning or classification methods which, to produce a robust model in the learning process, requires a substantial number of case and control samples to avoid the overfitting problem.

## Conclusions

We proposed a novel computational method sKLD solely based on a single case sample. This method can effectively detect the pre-disease state of complex diseases, a state with high susceptibility before the disease onset or deterioration. As the algorithm shown in Methods section, the sKLD is easy to implement and very flexible. It is therefore of great potential in personalized pre-disease diagnosis and prevention medicine. The identification of sKLD-signaling genes is also helpful in elucidating molecular mechanism of disease progression, and discovering prognosis indicators.

## Methods

### Theoretical background

The theoretical background is our recently proposed DNB theory. Specifically, in order to theoretically and mathematically describe the dynamics of a complex disease, its evolution is usually modeled as a time-dependent nonlinear dynamical system [23, 69], in which the sudden deterioration is regarded as a state transition at a bifurcation point [16]. In ideal situation with small noise, when a complex system is near the critical point, among all observed variables there exists a dominant group defined as the DNB biomolecules, which satisfy the following three conditions based on the observed data [10]:

- The correlation (PCC_in_) between any pair of members in the DNB group rapidly increases;
- The correlation (PCC_out_) between one member of the DNB group and any other non-DNB member rapidly decreases;
- The standard deviation (SD_in_) or coefficient of variation for any member in the DNB group drastically increases.

The above three properties are necessary conditions of the state transition at a codimension-one bifurcation point, and can also be approximately stated as: the occurrence of a group of biomolecules whose expressions are strongly fluctuating and highly correlated, implies an upcoming critical transition. These three properties are the theoretical basis of DNB method and have been proved in the supplementary information of our previous work [10].

From the above three properties, it is clear that the critical transition of a system is actually indicated by “the transition of distribution”, that is, for some variables (DNB members), their distribution would significantly change when the system approaches the critical transition point. Therefore, by exploring the differential distributions (rather than differential expressions) of some variables, it is possible to predict the upcoming qualitative state transition. On the other hand, a sample of high-throughput data enables us to analyze the expressions of thousands of genes simultaneously. Such a high-dimensional sample is actually enriched with dynamic information of accumulated effects, such as the gene interaction after a long-term development of the concerned biological system.

The Kullback–Leibler divergence (K–L divergence) was widely employed to measure the difference between two data distributions [70]. It provides a theoretical basis for data differencing [71], outlier detection [72] and evaluating sample similarity [73, 74]. Between two distributions *P* and *Q*, the K-L divergence is defined as

1 $$ {D}_{KL}\left(P,Q\right)=\sum \limits_{\mathrm{k}}\ln \left(\frac{P(k)}{Q(k)}\right)P(k). $$

It should be noted that the K-L divergence in Eq. (1) is actually not a true metric, but usually serves as a measure of the similarity between distributions *P* and *Q*. Particularly, *D*_*KL*_(*P*, *Q*) is zero only when the distribution *P* is identical with the distribution *Q*. *D*_*KL*_(*P*, *Q*) is positive when the distribution *P* is different from *Q*. Clearly, for the original K-L divergence, there is *D*_*KL*_(*P*, *Q*) ≠ *D*_*KL*_(*Q*, *P*). In this study, we use a symmetric measure defined as

2 $$ {D}_{KL}\left(P,Q\right)=\frac{\sum \limits_{\mathrm{k}}\ln \left(\frac{P(k)}{Q(k)}\right)P(k)+\sum \limits_{\mathrm{k}}\ln \left(\frac{Q(k)}{P(k)}\right)Q(k)}{2}. $$

### Algorithm to identify the tipping point based on sKLD

Regarding a biological system as a time-dependent nonlinear dynamical system with *m* genes/variables, then at each time point, the state of such system is expressed by a high-dimensional vector, i.e., the expressions of *m* genes/variables. A computational way is then developed to exploring the dynamic difference between the normal state and pre-disease state.

Given a set of reference samples (samples from normal cohort which are used as the background that represent the healthy or relatively healthy individuals), the following algorithm is proposed to identify the pre-disease state by using only one case sample.

[Step 1] Prepare a set of reference samples. The samples derived from the normal cohort are regarded as reference samples, which represent the background of relatively healthy individuals in the normal state. For numerical simulation, samples from a few initial time points are viewed as reference/normal samples. For real datasets, samples from a normal cohort or normal tissue are chosen as reference/normal samples, e.g., for the stage-course data from TCGA, the tumor-adjacent samples are taken as the reference.

[Step 2] Fit a distribution for each gene in terms of the expressions from the reference samples. Specifically, for a gene g_*i*_, a Gaussian distribution ${\mathrm{D}}_{{\mathrm{g}}_{\mathrm{i}}}$ is fitted based on the *k* expressions of g_*i*_ in the reference samples {*S*_1_, *S*_2_, …, *S*_k_}. Then, a *k*-dimensional vector ($\mathrm{area}\left({\mathrm{D}}_{{\mathrm{g}}_{\mathrm{i}}}\left({S}_1\right)\right)$, $\mathrm{area}\left({\mathrm{D}}_{{\mathrm{g}}_{\mathrm{i}}}\left({S}_2\right)\right)$,…, $\mathrm{area}\left({\mathrm{D}}_{{\mathrm{g}}_{\mathrm{i}}}\left({S}_k\right)\right)$) is obtained, in which the *j*-th element is the cumulative area (its definition was shown in the Eq. (S4) of Additional file 1) determined by the fitted distribution ${\mathrm{D}}_{{\mathrm{g}}_{\mathrm{i}}}$ and the expression of g_*i*_ in the *j*-th sample *S*_*j*_ (Fig. 1b).

[Step 3] Construct the reference distribution *P* as follows.

3 $$ P=\left[\begin{array}{c}{p}_1\\ {}{p}_2\\ {}\vdots \\ {}{p}_m\end{array}\right],{p}_i=\frac{\mathrm{mean}\left[ area\left({\mathrm{D}}_{\mathrm{g}i}\left({S}_1\right)\right)\kern0.5em area\left({\mathrm{D}}_{\mathrm{g}i}\left({S}_2\right)\right)\kern0.5em \cdots \kern0.5em area\left({\mathrm{D}}_{\mathrm{g}i}\left({S}_k\right)\right)\right]}{\sum \limits_{j=1}^m\mathrm{mean}\left[ area\left({\mathrm{D}}_{gj}\left({S}_1\right)\right)\kern0.5em area\left({\mathrm{D}}_{gj}\left({S}_2\right)\right)\kern0.5em \cdots \kern0.5em area\left({\mathrm{D}}_{gj}\left({S}_k\right)\right)\right]},\kern1em i=1,2,...,m. $$

[Step 4] For a single case sample s_case_ of an individual, construct a perturbed distribution *Q* based on s_case_ as follows.

4 $$ Q=\left[\begin{array}{c}{q}_1\\ {}{q}_2\\ {}\vdots \\ {}{q}_m\end{array}\right],{q}_i=\frac{area\left({\mathrm{D}}_{\mathrm{g}i}\left({S}_{\mathrm{case}}\right)\right)}{\sum \limits_{j=1}^m area\left({D}_{gj}\left({S}_{\mathrm{case}}\right)\right)},\kern1em i=1,2,...,m. $$

For both distributions *P* and *Q*, it is clear that $\sum \limits_{j=1}^m{p}_j=1$ and $\sum \limits_{j=1}^m{q}_j=1$.

[Step 5] Calculate the sKLD score based on Eq. (2). Clearly, such score evaluated the difference between the reference distribution *P* and the perturbed distribution *Q*.

According to the DNB theory, when the system approaches the critical state, the DNB biomolecules exhibit significantly collective behaviors with fluctuations (see the supplementary information of reference [18] for detailed derivation in the ideal situation), which leads to that the distributions of DNB genes in a pre-disease state are different from those in a normal state. Thus, the background distribution from a set of reference/normal samples significantly distinct to the perturbed distribution from a new case sample, leading to the increase of sKLD score in Eq. (2). Thus, sKLD score can provide the early-warning signals of the critical transition. From above algorithm, it is seen that the proposed method is data-driven, and thus model free.

### Data processing and functional analysis

The proposed method has been applied to six real datasets, i.e., the time-course dataset GSE2565 from NCBI GEO database (http://www.ncbi.nlm.nih.gov/geo) and five stage-course datasets LUSC, LUAD, STAD, THCA and COAD from TCGA database (http://cancergenome.nih.gov). The omics dataset GSE2565 comprises expression profiles from a mouse experiment, in which pulmonary edema was triggered by inhalation of carbonyl chloride. In this dataset, we discarded the probes without corresponding NCBI Entrez gene symbol. For each gene mapped by multiple probes, the average value was employed as the gene expression. The five stage-course datasets from TCGA contained RNA-Seq data and included both tumor and tumor-adjacent samples. The tumor samples were divided into different stages based on clinical (stage) information from TCGA, and the samples without stage information were ignored.

For all the diseases, functional annotations were performed by searching the NCBI gene database (http://www.ncbi.nlm.nih.gov/gene). The enrichment analyses were separately obtained using web service tools from the Gene Ontology Consortium (GOC, http://geneontology.org) and client software from Ingenuity Pathway Analysis (IPA, http://www.ingenuity.com/products/ipa).

## Supplementary information

**Additional file 1.** Identifying critical state by single-sample Kullback–Leibler divergence.

**Additional file 2.** The signaling genes of LUSC, LUAD, STAD, THCA and COAD.

**Additional file 3.** The common signaling genes for acute lung injury.

**Additional file 4.** The common sKLD-signaling genes for LUSC, LUAD and STAD.

## Data Availability

Lung squamous cell carcinoma (LUSC), lung adenocarcinoma (LUAD), stomach adenocarcinoma (STAD), thyroid carcinoma (THCA) and colon adenocarcinoma (COAD) are available from the cancer genome atlas (TCGA) database (http://cancergenome.nih.gov). Acute lung injury (GSE2565) is available from NCBI GEO database (http://www.ncbi.nlm.nih.gov/geo). The source code of algorithm is accessed in (https://github.com/zhongjiayuna/KL_Project).

## Abbreviations

COAD: Colon adenocarcinoma
DNB: The dynamical network biomarker
LUAD: Lung adenocarcinoma
LUSC: Squamous cell carcinoma
PCC: Pearson correlation coefficient
SD: Standard deviation
sKLD: Single-sample Kullback–Leibler divergence
STAD: Stomach adenocarcinoma
TCGA: The cancer genome atlas
THCA: Thyroid carcinoma
