# Supplementary material for: Identifying critical state of complex diseases by single-sample Kullback–Leibler divergence
Source: BMC Genomics. 2020 Jan 28;21:87. doi: 10.1186/s12864-020-6490-7 (PMC6988219; doi:10.1186/s12864-020-6490-7)
Supplement: Supplementary file 1 — Additional file 1. Identifying critical state by single-sample Kullback–Leibler divergence. [file 12864_2020_6490_MOESM1_ESM.docx]

**Supplementary Information: Identifying critical state by single-sample Kullback–Leibler divergence**

**Contents**

**A. The detailed dynamical system theory about numerical simulation** S2

**B. Validating the identified critical state by The Kaplan-Meier (log-rank) survival analysis** S5

B1. A schematic for validating the identified critical state S5

B2. Validating the identified critical state for five tumor disease S6

**C. The functional analysis for THCA and CAOD** S9

C1. The functional analysis for THCA S9

C2. The functional analysis for COAD S12

C3. The application of sKLD in influenza infection dataset S14

1. **The detailed dynamical system theory about numerical simulation**

We used a regulatory network with 8 genes (see Fig. 3A in the main text) to conduct a numerical simulation for detecting the pre-disease state using sKLD. These types of gene molecular regulatory networks are often used to study various biological processes including transcription, translation, diffusion, and translocation processes that affect gene regulatory activities (1-5). The following 8 differential equations represent the gene regulation of 8 genes in a network. In network, gene regulation is represented in a Michaelis-Menten form with the degradation rates, which are linearly proportional to the concentrations of the corresponding genes.

$$\left\{\begin{matrix} \frac{\text{d}z_{1}(t)}{dt}=\frac{(4-3|s|)z_{2}(t)}{10(1+z_{2}(t))}-\frac{(4+3|s|)}{10}z_{1}(t)+\zeta_{1}(t) \\ \frac{\text{d}z_{2}(t)}{dt}=\frac{(4-3|s|)z_{1}(t)}{10(1+z_{1}(t))}-\frac{(4+3|s|)z_{2}(t)}{10(1+z_{2}(t))}+\zeta_{2}(t) \\ \frac{\text{d}z_{3}(t)}{dt}=\frac{(6|s|-10)}{10}+\frac{(5-3|s|)}{10(1+z_{1}(t))}+\frac{(5-3|s|)}{10(1+z_{2}(t))}-z_{3}(t)+\zeta_{3}(t) \\ \frac{\text{d}z_{4}(t)}{dt}=\frac{(6|s|-12)}{10}+\frac{(6-3|s|)z_{1}(t)}{10(1+z_{1}(t))}+\frac{(6-3|s|)z_{2}(t)}{10(1+z_{2}(t))}-\frac{6}{5}z_{4}(t)+\zeta_{4}(t) \\ \frac{\text{d}z_{5}(t)}{dt}=\frac{(6|s|-14)}{10}+\frac{(7-3|s|)z_{1}(t)}{10(1+z_{1}(t))}+\frac{(7-3|s|)z_{2}(t)}{10(1+z_{2}(t))}-\frac{7}{5}z_{5}(t)+\zeta_{5}(t) \\ \frac{\text{d}z_{6}(t)}{dt}=-\frac{3}{5}+\frac{1}{10(1+z_{1}(t))}+\frac{1}{10(1+z_{2}(t))}+\frac{1}{5(1+z_{5}(t))}+\frac{1}{5(1+z_{7}(t))} \\ +\frac{z_{8}(t)}{5(1+z_{8}(t))}-\frac{8}{5}z_{6}(t)+\zeta_{6}(t) \\ \frac{\text{d}z_{7}(t)}{dt}=\frac{z_{8}(t)}{10(1+z_{8}(t))}-\frac{19}{10}z_{7}(t)+\zeta_{7}(t) \\ \frac{\text{d}z_{8}(t)}{dt}=\frac{z_{7}(t)}{10(1+z_{7}(t))}-\frac{19}{10}z_{8}(t)+\zeta_{8}(t) \end{matrix}\right.$$

$$\left(S\right.\left.1\right)$$

where $s$ is a scalar control parameter and $\varsigma_{i}(t)$(*i* = 1, 2, ..., 8) are Gaussian noises with zero means and covariances $\text{k}_{ij}=Cov(\varsigma_{i},\varsigma_{j})$. $z_{i}(t)$(*i* = 1, 2, ..., 8) represent the concentrations of mRNA-i. In Eq.(S1), there is the degradation rates of mRNAs $\text{R=(}\frac{(4+3|s|)}{10}\text{,}\frac{(4+3|s|)}{10}\text{,1,}\frac{6}{5}\text{,}\frac{7}{5}\text{,}\frac{8}{5}\text{,}\frac{19}{10}\text{,}\frac{19}{10}\text{).}$The stable equilibrium point of the differential equations Eq.(S1) is $\overline{Z}=(\overline{z_{1}},\overline{z_{2}},\overline{z_{3}},\ldots,\overline{z_{8}})=(0,0,0,\ldots,0)$. The differential equations Eq.(S1) can be transformed into the difference equations $Z(k+1)=f(Z(k),S)$using the Euler scheme (6) with a small time interval $\Delta t$. The result is as follows:

$$\left\{\begin{matrix} z_{1}(k+1)=z_{1}(k)+\left[\frac{(4-3|s|)z_{2}(t)}{10(1+z_{2}(t))}-\frac{(4+3|s|)}{10}z_{1}(t)+\zeta_{1}(t)\right]\Delta t \\ z_{2}(k+1)=z_{2}(k)+\left[\frac{(4-3|s|)z_{1}(t)}{10(1+z_{1}(t))}-\frac{(4+3|s|)z_{2}(t)}{10(1+z_{2}(t))}+\zeta_{2}(t)\right]\Delta t \\ z_{3}(k+1)=z_{3}(k)+\left[\frac{(6|s|-10)}{10}\text{+}\frac{(5-3|s|)}{10(1+z_{1}(t))}+\frac{(5-3|s|)}{10(1+z_{2}(t))}-z_{3}(t)+\zeta_{3}(t)\right]\Delta t \\ z_{4}(k+1)=z_{4}(k)+\left[\frac{(6|s|-12)}{10}+\frac{(6-3|s|)z_{1}(t)}{10(1+z_{1}(t))}+\frac{(6-3|s|)z_{2}(t)}{10(1+z_{2}(t))}-\frac{6}{5}z_{4}(t)+\zeta_{4}(t)\right]\Delta t \\ z_{5}(k+1)=z_{5}(k)\text{+}\left[\frac{(6|s|-14)}{10}+\frac{(7-3|s|)z_{1}(t)}{10(1+z_{1}(t))}+\frac{(7-3|s|)z_{2}(t)}{10(1+z_{2}(t))}-\frac{7}{5}z_{5}(t)+\zeta_{5}(t)\right]\Delta t \\ z_{6}(k+1)=z_{6}(k)+\left[-\frac{3}{5}+\frac{1}{10(1+z_{1}(t))}+\frac{1}{10(1+z_{2}(t))}+\frac{1}{5(1+z_{5}(t))}+\frac{1}{5(1+z_{7}(t))}\right. \\ \left.+\frac{z_{8}(t)}{5(1+z_{8}(t))}-\frac{8}{5}z_{6}(t)+\zeta_{6}(t)\right]\Delta t \\ z_{7}(k+1)=z_{7}(k)+\left[\frac{z_{8}(t)}{10(1+z_{8}(t))}-\frac{19}{10}z_{7}(t)+\zeta_{7}(t)\right]\Delta t \\ z_{8}(k+1)=z_{8}(k)+\left[\frac{z_{7}(t)}{10(1+z_{7}(t))}-\frac{19}{10}z_{8}(t)+\zeta_{8}(t)\right]\Delta t \end{matrix}\right.$$

$$\left(S2\right)$$

It is easy to note that $Z\text{（}k)$ is the vector of $Z\text{（}t\text{）}$ at the time instant $k\Delta t$. The Jacobian matrix of Eq.(S2) can be defined as $\left.J\text{=}\frac{\partial f(Z(k);S)}{\partial Z}\right|_{Z=\overline{Z}}$, where

$$J=e^{\Delta t\bullet A}$$

$$\left(S3\right)$$

From Eq.(S3), by taking $\Delta t=1$, we can obtain eight distinct eigenvalues with the largest eigenvalue 0.66$\left|s\right|$→ 1 when $s$→ 0. Therefore, the equilibrium point $\overline{Z}$is stable when $s\in(0,1]$ and $S_{c}=0$ is a critical value, at which the system undergoes a critical transition. Based on the theoretical model Eq.(S2), we collected time-course data of the 8-gene expressions under each parameter condition. Then, we simulated the mean sKLD curve, as shown in Fig.2b in the main text.

Besides, by using the simulated dataset generated from Eq. (S1), we have the following discussion. As shown in Figure S1, we have analyzed the signals when the number of reference samples varies. It can be seen from Figure S1 that the sKLD index accurately indicates the tipping point under different reference sample sizes. Therefore, different settings of the number of reference samples do not affect the evolution tendency of the signal curve.


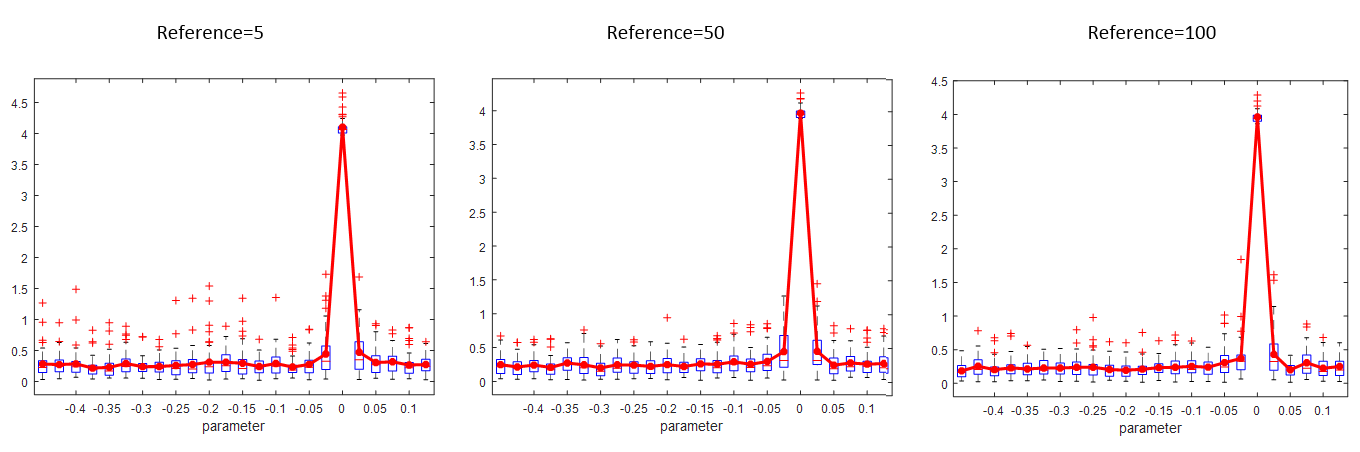


Figure S1: The critical signals under different reference sample sizes.

As shown in Figure S2, we have analyzed the signals when noise strength $\sigma$varies. It can be seen from Figure S2 that although the strength of the signal may change, the sKLD index accurately indicates the tipping point with a similar tendency. Therefore, different settings of noise strength $\sigma$ may affect the strength of the signal, but do not affect the evolution tendency (e.g., abrupt increase when approaching the tipping point) of the signal curve.


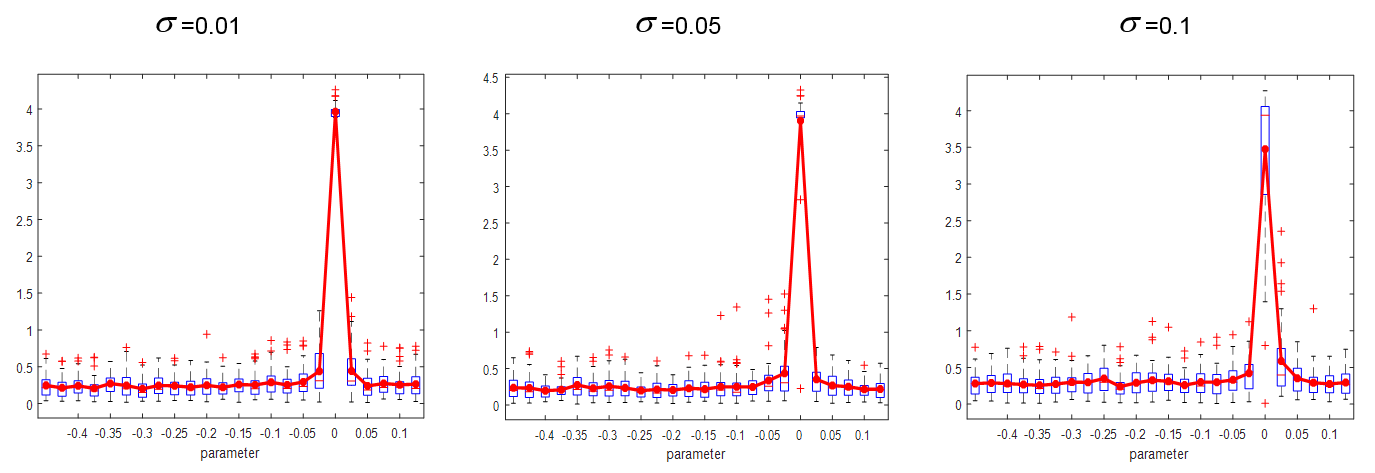


Figure S2: The critical signals under different noise strength.

**The definition of "cumulative area"**

For the simulated Gaussian distribution $N(\mu,\sigma)$ of a gene $\text{g}_{i}$, where $\mu$ is the mean and $\sigma$ is the standard deviation, the "cumulative area" $D_{gi}(x_{gi})$ is defined as Eq. (S4),

$$D_{gi}(x_{gi})=\int_{-\infty}^{x_{gi}} d_{gi}(s)ds$$

$$\left(S4\right)$$

where $x_{gi}$ represents the gene expression of $\text{g}_{i}$, and $d_{gi}$ is the probability density function. An illustrative process of calculating $D_{gi}(x_{gi})$ is shown in Fig. S3 below


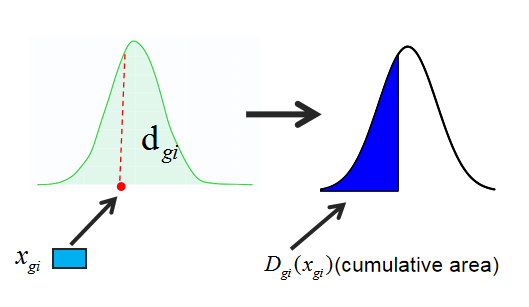


Figure S3: An illustrative process about the calculation of "cumulative area".

1. **Validating the identified critical state by The Kaplan-Meier (log-rank) survival analysis**

**B1. A schematic for validating the identified critical state**

To validate the identified critical state, the prognosis results respectively based on before-transition and after-transition samples were exhibited and compared through Kaplan-Meier (log-rank) survival analysis. For instance, the following four steps (survival test 1, survival test 2, survival test 3 and survival test 4) were carried out to validate a critical transition of tumor disease at stage IIA. Specifically, the survival time of before-transition samples is much longer than that of after-transition samples. However, before and after any other stages, there was no significant difference in the survival time. A schematic illustration for validating the identified critical state is shown in Figure S4.

**
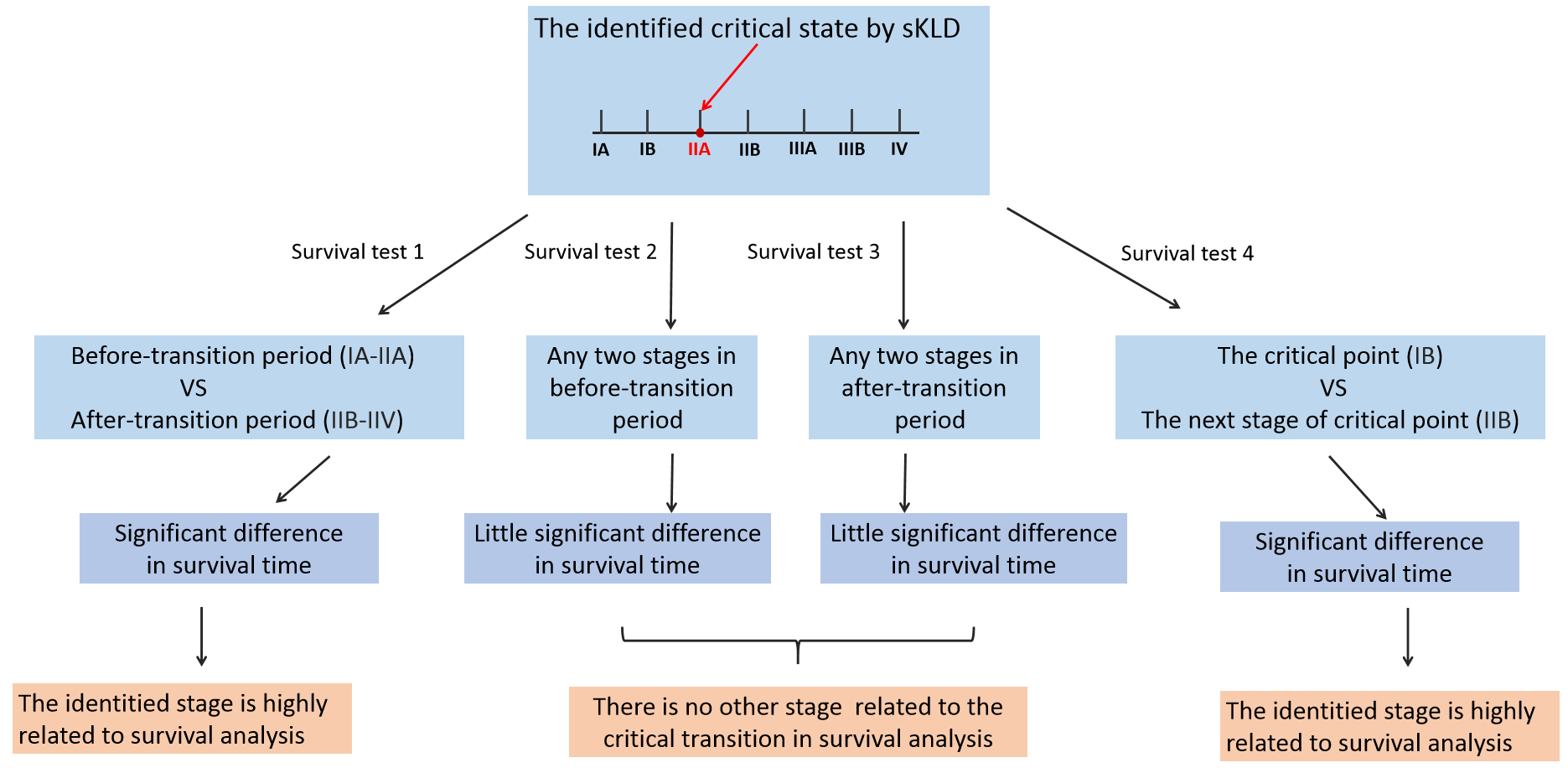
**

Figure S4: A schematic illustration for validating the identified critical state.

**B2. Validating the identified critical state for five tumor disease**

In this study, our method is applied to five tumor datasets, lung squamous cell carcinoma (LUSC), lung adenocarcinoma (LUAD), stomach adenocarcinoma (STAD), thyroid carcinoma (THCA), colon adenocarcinoma (COAD) from the cancer genome atlas (TCGA). There are three criteria for the dataset selection: 1, they are all five stages datasets, which indicate the state of the cancer; 2, both tumor and tumor-adjacent samples are available in these datasets; 3, clinical information is available in all these datasets so as to carry out the survival analysis. Each tumor dataset was composed by tumor and tumor-adjacent samples. The tumor samples were grouped into different cancer stages according to corresponding clinical information of TCGA. Specifically, the tumor samples were classified into seven stages (stage IA, IB, IIA, IIB, IIIA, IIIB, and IV) for LUSC, LUAD and STAD. The tumor samples were classified into four stages (stage I, II, III, and IV) for THCA and COAD. The detailed number of samples within each stage are shown as the following table S1.

**Table S1 Number of samples within each stage in tumor disease dataset from TCGA.**


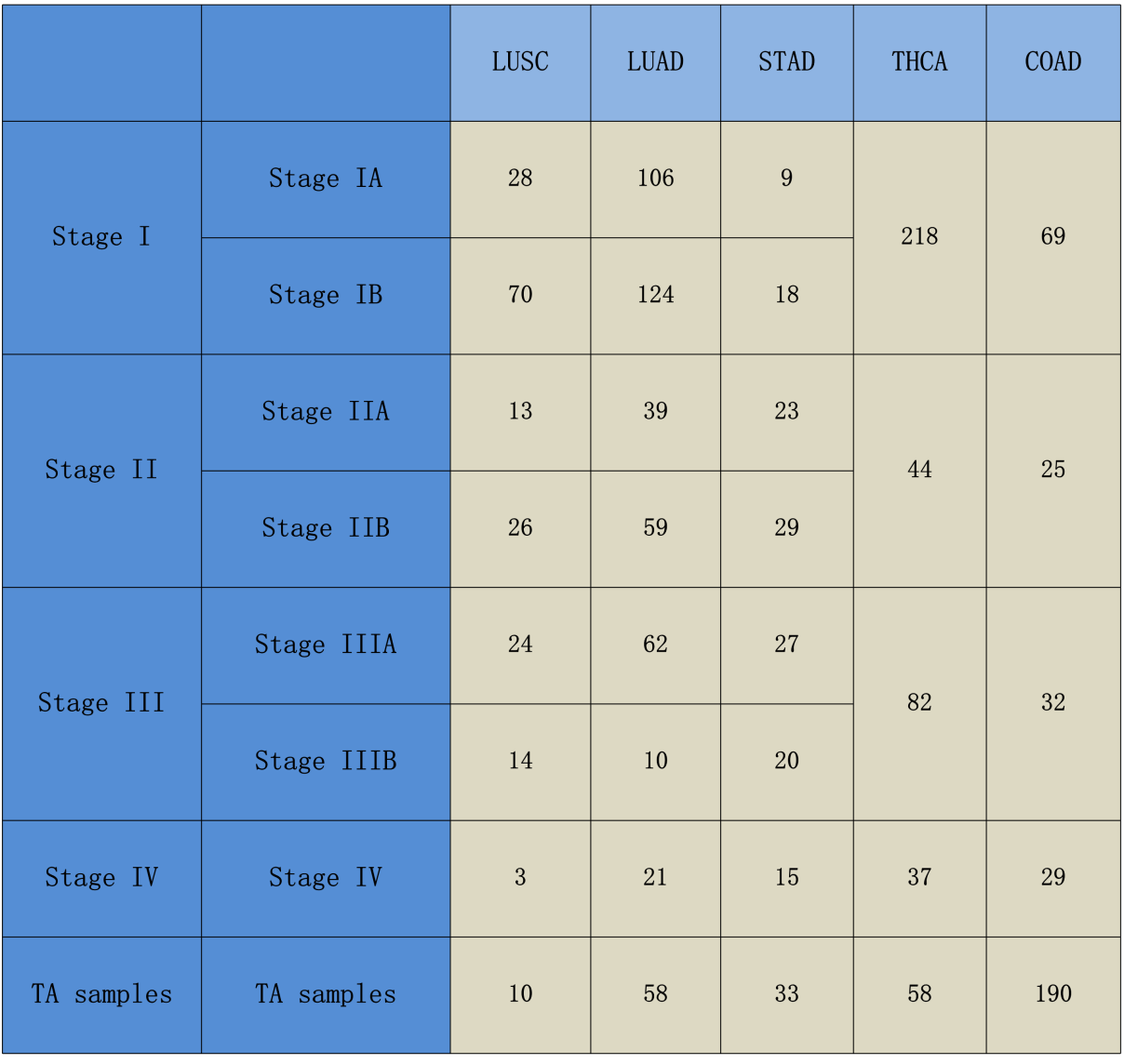


To illustrate the validity of the critical state identified by sKLD, the Kaplan-Meier (log-rank) survival analysis was conducted based on before-transition and after-transition samples. The results were shown in the following Figure S5. The studies consistently showed that the survival time of samples from the before-transition period is much longer than that from after-transition period. Besides, there is little significant difference among the survival time of samples from the stages before the critical state and little difference among the survival time of samples from the stages after the critical state. These results demonstrated that the sKLD method can detect the early-warning signals of a critical transition of survive, which validated the identified critical state identified by sKLD.


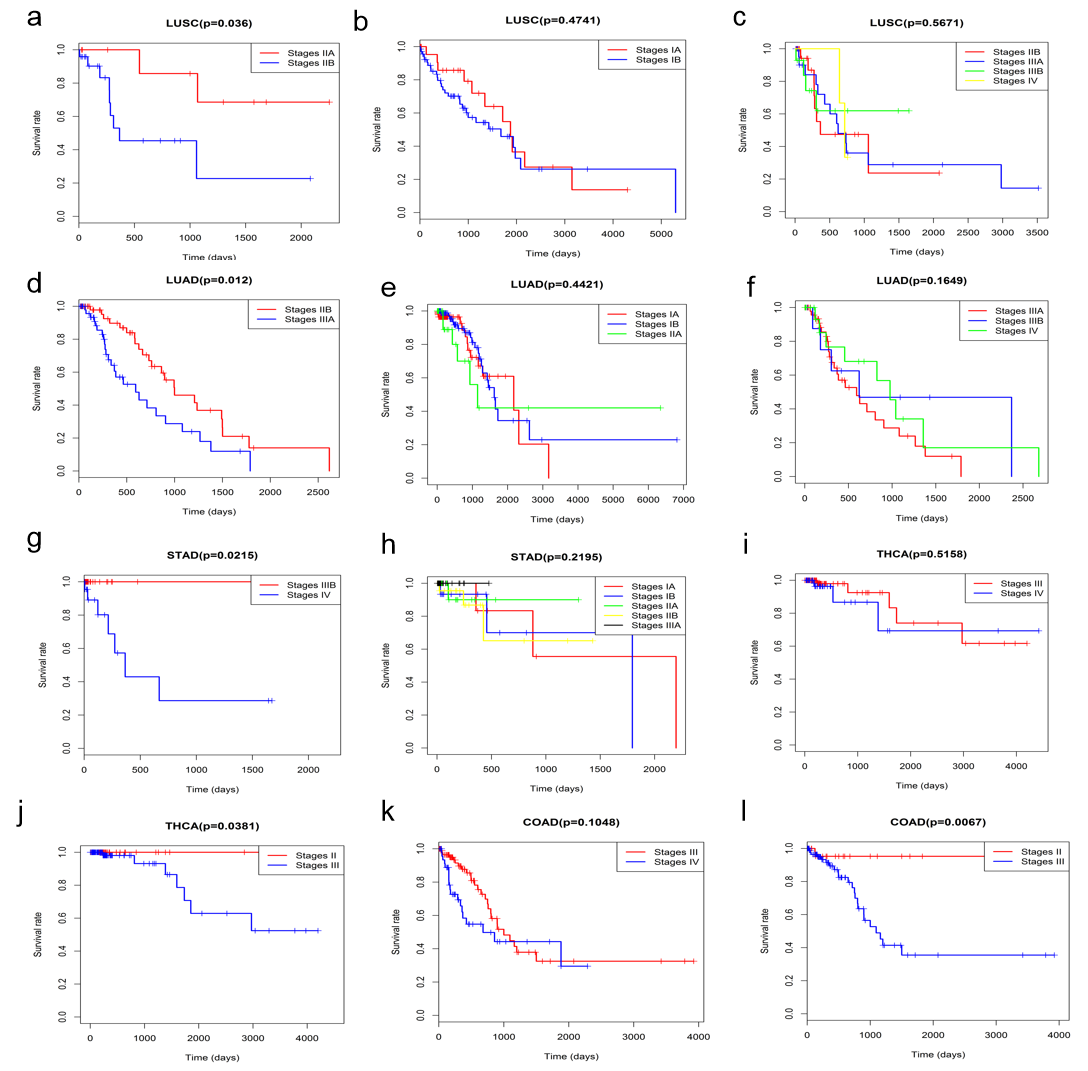


Figure S5: **a** The critical point (IIA) of LUSC vs the next stage of critical point (IIB) of LUSC in survival analysis. **b** Two stages in before-transition period (IA-IB) of LUSC in survival analysis. **c** Any two stages in after-transition period (IIB-IV) of LUSC in survival analysis. **d** The critical point (IIB) of LUAD vs the next stage of critical point (IIIA) of LUAD in survival analysis. **e** Any two stages in before-transition period (IA-IIA) of LUAD in survival analysis. **f** Any two stages in after-transition period (IIIA-IV) of LUAD in survival analysis. **g** The critical point (IIIB) of STAD vs the next stage of critical point (IV) of STAD in survival analysis. **h** Any two stages in before-transition period (IA-IIIA) of STAD in survival analysis. **i** Two stages in after-transition period (III-IV) of THCA in survival analysis. **j** The critical point (II) of THCA vs the next stage of critical point (III) of THCA in survival analysis. **k** Two stages in after-transition period (III-IV) of COAD in survival analysis. **I** The critical point (II) of COAD vs the next stage of critical point (III) of COAD in survival analysis.

We also carried out the survival analysis for the signaling genes of LUSC, LUAD, and THCA. As shown in Figure S6, for LUSC, the survival rate of patients obviously decreased when genes such as B4GALNT4, POLR2J3, and NFKBIL2 expressed abnormally. For LUAD, the survival rate of patients significantly decreased when the genes such as ARSH, NR2E1, and SFXN1 expressed abnormally. For THCA, the survival rate of patients significantly decreased when the genes such as PPP1R15B, CTSD, and DCN expressed abnormally.


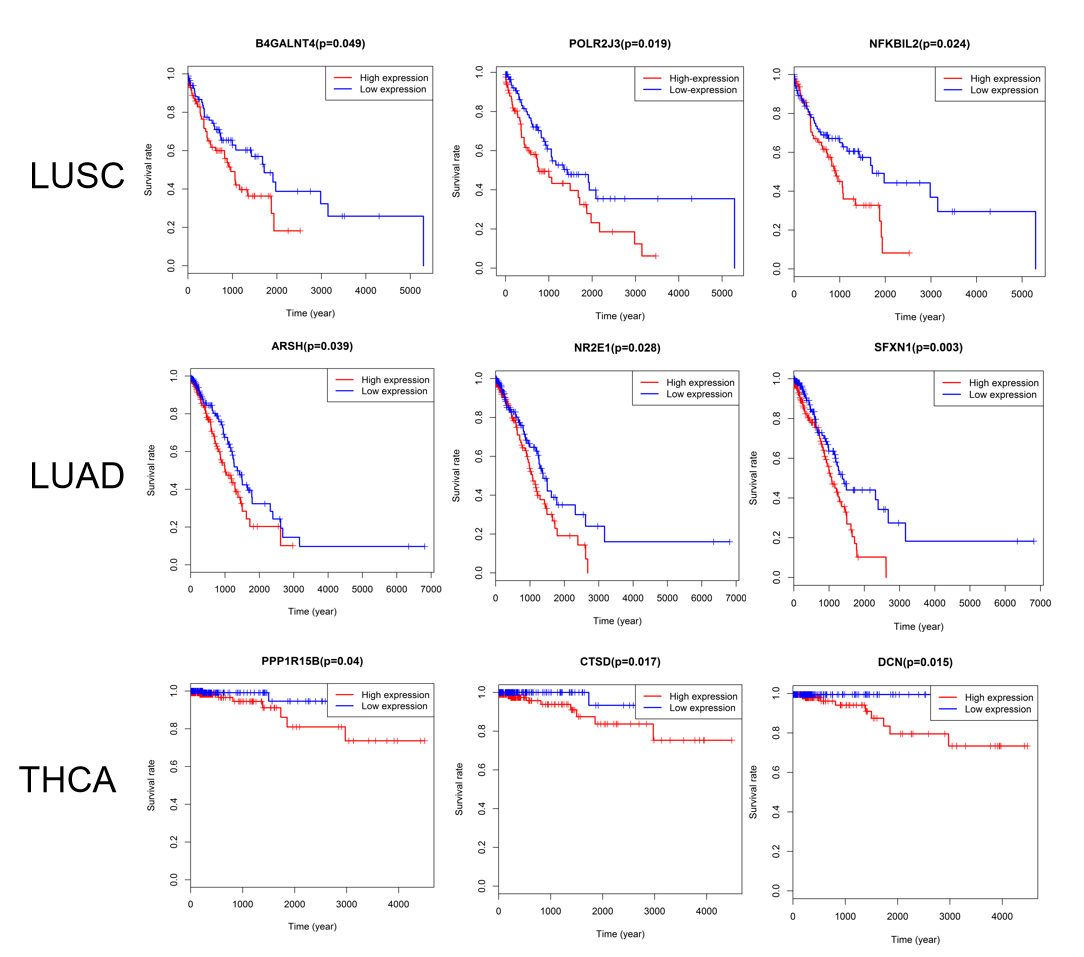


Figure S6: Comparison of survival curves for the signaling genes of LUSC, LUAD and THCA.

**C. The functional analysis for THCA and CAOD**

**C1. The functional analysis for THCA**

Some “sKLD-signaling genes” have been shown to be associated with the process of cancer THCA through literature searching (S2 Table). For example, FCGBP expression is reduced in thyroid cancer, which might suggest FCGBP plays an important role in thyroid cell division and migration [7]. Methylation of GPX3 is related to tumor size and lymph node metastasis in human thyroid cancer and GPX3 can suppress metastasis of thyroid cancer through inhibition Wnt/β-catenin signaling [8]. The high expression of GABRB2 likely induces more lymph node metastasis in thyroid carcinoma [9]. MiR-204-5p can suppress cell proliferation by inhibiting IGFBP5 in papillary thyroid carcinoma [10]. TIMP3 can regulate migration, invasion and in vivo tumorigenicity of thyroid tumor cells [11]. METTL7B can promote migration and invasion in thyroid cancer through epithelial-mesenchymal transition [12]. High expression of NEAT1 promoted the onset of thyroid carcinoma. In addition, NEAT1 promoted the malignant progression of thyroid cancer through regulating miRNA-214 expression [13]. LRIG1 functions as a thyroid tumor suppressor and plays an important role in the progression of thyroid cancer [14].

Besides, through GO analysis, the common “sKLD-signaling genes” are highly related to functions annotation, such as thyroid hormone generation, signal transduction, nervous system development and others (S3 Table). These biological processes are associated with the progression of cancer. Moreover, according to IPA, the common “sKLD-signaling genes” are associated with thyroid gland tumor, thyroid cancer, cell proliferation of tumor cell lines, etc. (S3 Table).

**Table S2 Genes with high frequency in 44 “sKLD-signaling genes” groups in the critical stage (stage II) for THCA.**

| **Gene** | **Frequency** | **Location** | **Family*** | Relation with cancer progression |
| --- | --- | --- | --- | --- |
| FCGBP | 41 | Extracellular Space | other | FCGBP expression is reduced in thyroid cancer, which might suggest FCGBP plays an important role in thyroid cell division and migration [1]. |
| GPX3 | 41 | Extracellular Space | enzyme | Methylation of GPX3 is related to tumor size and lymph node metastasis in human thyroid cancer and GPX3 can suppress metastasis of thyroid cancer through inhibition Wnt/β-catenin signaling [2]. |
| GABRB2 | 40 | Plasma Membrane | ion channel | The high expression of GABRB2 likely induces more lymph node metastasis in thyroid carcinoma [3]. |
| IGFBP5 | 39 | Extracellular Space | other | MiR-204-5p can suppress cell proliferation by inhibiting IGFBP5 in papillary thyroid carcinoma [4]. |
| TIMP3 | 38 | Extracellular Space | other | TIMP3 can regulate migration, invasion and in vivo tumorigenicity of thyroid tumor cells [5]. |
| METTL7B | 38 | Other | enzyme | METTL7B can promote migration and invasion in thyroid cancer through epithelial-mesenchymal transition [6]. |
| NEAT1 | 37 | Other | other | High expression of NEAT1 promoted the onset of thyroid carcinoma. In addition, NEAT1 promoted the malignant progression of thyroid cancer through regulating miRNA‑214 expression [7]. |
| LRIG1 | 37 | Extracellular Space | other | LRIG1 functions as a thyroid tumor suppressor and plays an important role in the progression of thyroid cancer [8]. |

**Table S3 Functional enrichment of common “sKLD-signaling genes” in the critical stage samples for THCA.**

| **Gene Ontology Consortium** | | **IPA** | |
| --- | --- | --- | --- |
| **enriched biological process** | **enriched *p* value** | **enriched biological process** | **enriched *p* value** |
| thyroid hormone generation (GO:0006590) | 9.21E-07 | thyroid gland tumor | 4.31E-33 |
| signal transduction (GO:0007165) | 1.66E-06 | thyroid cancer | 7.71E-32 |
| nervous system development (GO:0007399) | 7.07E-05 | cell proliferation of tumor cell lines | 8.24E-17 |
| cell death (GO:0008219) | 2.91E-04 | differentiated thyroid cancer | 2.08E-12 |
| extracellular matrix organization (GO:0030198) | 6.02E-04 | papillary thyroid carcinoma | 3.40E-12 |
| transmembrane transport (GO:0055085) | 9.64E-04 | abnormal morphology of thyroid gland | 3.80E-09 |

**C2. The functional analysis for COAD**

Through literature searching, some genes in the common “sKLD-signaling genes” have been identified to be associated with the process of COAD (S4 Table). For instance, Targeting CD46 in colon cancer cells may substantially suppress the pathogenesis and progression of the disease [15]. The high expression of EIF3E in colon cancer was significantly correlated with lymph node involvement and vessel invasion [16]. Knockdown of RPL9 expression inhibits colorectal carcinoma growth via the inactivation of Id-1/NF-kB signaling axis [17]. HMGB1 can attenuate anti-metastatic defense of the lymph nodes in colorectal cancer [18]. CD164 can promote colon cancer cell proliferation and metastasis both in vitro and in vivo and may be a new target for diagnosis and treatment for colon cancer [19]. The decreased EPCAM expression was found to be significantly linked to features of tumor invasion in colorectal cancer, including presence of lymph node metastasis and infiltrating tumor margin [20]. P53-induced miR-30e-5p can inhibit colorectal cancer invasion and metastasis by targeting ITGB1 [21]. S100A6 can stimulate proliferation and migration of colorectal carcinoma cells through activation of the MAPK pathways [22].

Besides, from GO analysis, the common “sKLD-signaling genes” are enriched in the biological processes including regulation of immune system process, defense response, positive regulation of T cell mediated cytotoxicity and others (S5 Table). All these biological processes are related with the progression of cancer. Furthermore, these common genes are also related to adhesion of colorectal cancer cell lines, colorectal disorder, and colorectal cancer (S5 Table) by functional enrichment from IPA.

**Table S4 Genes with high frequency in 13 “sKLD-signaling genes” groups in the critical stage (stage II) for COAD.**

| **Gene** | **Frequency** | **Location** | **Family*** | **Relation with cancer progression** |
| --- | --- | --- | --- | --- |
| CD46 | 25 | Cytoplasm | other | Targeting CD46 in colon cancer cells may substantially suppress the pathogenesis and progression of the disease [1]. |
| EIF3E | 25 | Cytoplasm | other | The high expression of EIF3E in colon cancer was significantly correlated with lymph node involvement and vessel invasion [2]. |
| RPL9 | 25 | Nucleus | other | Knockdown of RPL9 expression inhibits colorectal carcinoma growth via the inactivation of Id-1/NF-kB signaling axis [3]. |
| HMGB1 | 24 | Nucleus | transcription regulator | CD164 can promote colon cancer cell proliferation and metastasis both in vitro and in vivo and may be a new target for diagnosis and treatment for colon cancer [4]. |
| CD164 | 24 | Plasma Membrane | other | TIMP3 can regulate migration, invasion and in vivo tumorigenicity of thyroid tumor cells [5]. |
| EPCAM | 24 | Plasma Membrane | other | The decreased EPCAM expression was found to be significantly linked to features of tumor invasion in colorectal cancer, including presence of lymph node metastasis and infiltrating tumor margin [6]. |
| TGB1 | 24 | Plasma Membrane | transmembrane receptor | P53-induced miR-30e-5p can inhibit colorectal cancer invasion and metastasis by targeting ITGB1 [7]. |
| S100A6 | 24 | Cytoplasm | transporter | S100A6 can stimulate proliferation and migration of colorectal carcinoma cells through activation of the MAPK pathways [8]. |

**Table S5 Functional enrichment of common “sKLD-signaling genes” in the critical stage samples for COAD.**

| **Gene Ontology Consortium** | | **IPA** | |
| --- | --- | --- | --- |
| **enriched biological process** | **enriched *p* value** | **enriched biological process** | **enriched *p* value** |
| extracellular matrix organization (GO:0030198) | 3.32E-14 | digestive organ tumor | 4.04E-51 |
| collagen fibril organization (GO:0030199) | 5.31E-13 | digestive system cancer | 1.63E-50 |
| ribosome biogenesis (GO:0042254) | 1.08E-09 | abdominal cancer | 2.07E-49 |
| regulation of cell cycle (GO:0051726) | 1.61E-08 | gastrointestinal tract cancer | 7.11E-45 |
| collagen metabolic process (GO:0032963) | 3.67E-05 | gastrointestinal carcinoma | 1.49E-44 |
| regulation of protein ubiquitination (GO:0031396) | 9.60E-05 | development of digestive organ tumor | 5.90E-27 |

**C3. The application of sKLD in influenza infection dataset**

Dataset GSE30550 was generated from a human challenge of live influenza infection. The time-course dataset for influenza virus infection process (GSE30550) can be downloaded from the NCBI GEO database (www.ncbi.nlm.nih.gov/geo). For omics data (GSE30550), we discarded the probes without corresponding NCBI Entrez gene symbol. After removing any redundancy in dataset GSE30550, we obtained 11,451 molecules through probe mapping. For each gene mapped by multiple probes, the average value was employed as the gene expression. The gene expression profiles were derived in the whole peripheral blood drawn from all subjects at 16 time points, i.e., -24 hours before injection 0, 5, 12, 21, 29, 36, 45, 53, 60, 69, 77, 84, 93, 101, 108 hours after the injection. At each time point, there was only a single sample for each subject. We applied the proposed method to the time-course dataset of live influenza infection (GSE30550), in which there were seventeen subjects who received injection of influenza virus (H3N2/Wisconsin). Among the seventeen subjects, nine were infected who showed clinic symptoms including stuffy nose, scratchy throat, headache, cough, fever, myalgias etc. The information of these nine symptomatic subjects (Patients 1, 2, 3, 4, 5, 6, 7, 8, 9) was shown in Fig. S7B. For each symptomatic subject, the gene expression profiles of the initial 5 timepoints, i.e., -24 h, 0 h, 5 h,12 h and 21h, were regarded as individual reference samples, which represent the background of relatively healthy stage during the challenge. To determine whether there was a signal for the increase of sKLD score, a threshold was generated based on the ROC value. This computational technique was provided by the reference [23]. By employing the proposed method, the individual-specific sKLD scores for 9 symptomatic subjects were shown in Figure S7A. For each sKLD curve, the blue star represented the time point when sKLD signal arose, while the purple circle represented the time point at which the initial flu symptoms appeared. The sKLD score indicated the pre-disease states (the state before the appearance of flu infection symptoms) for these nine symptomatic subjects, with 89% accuracy (eight successful detections and one failure).


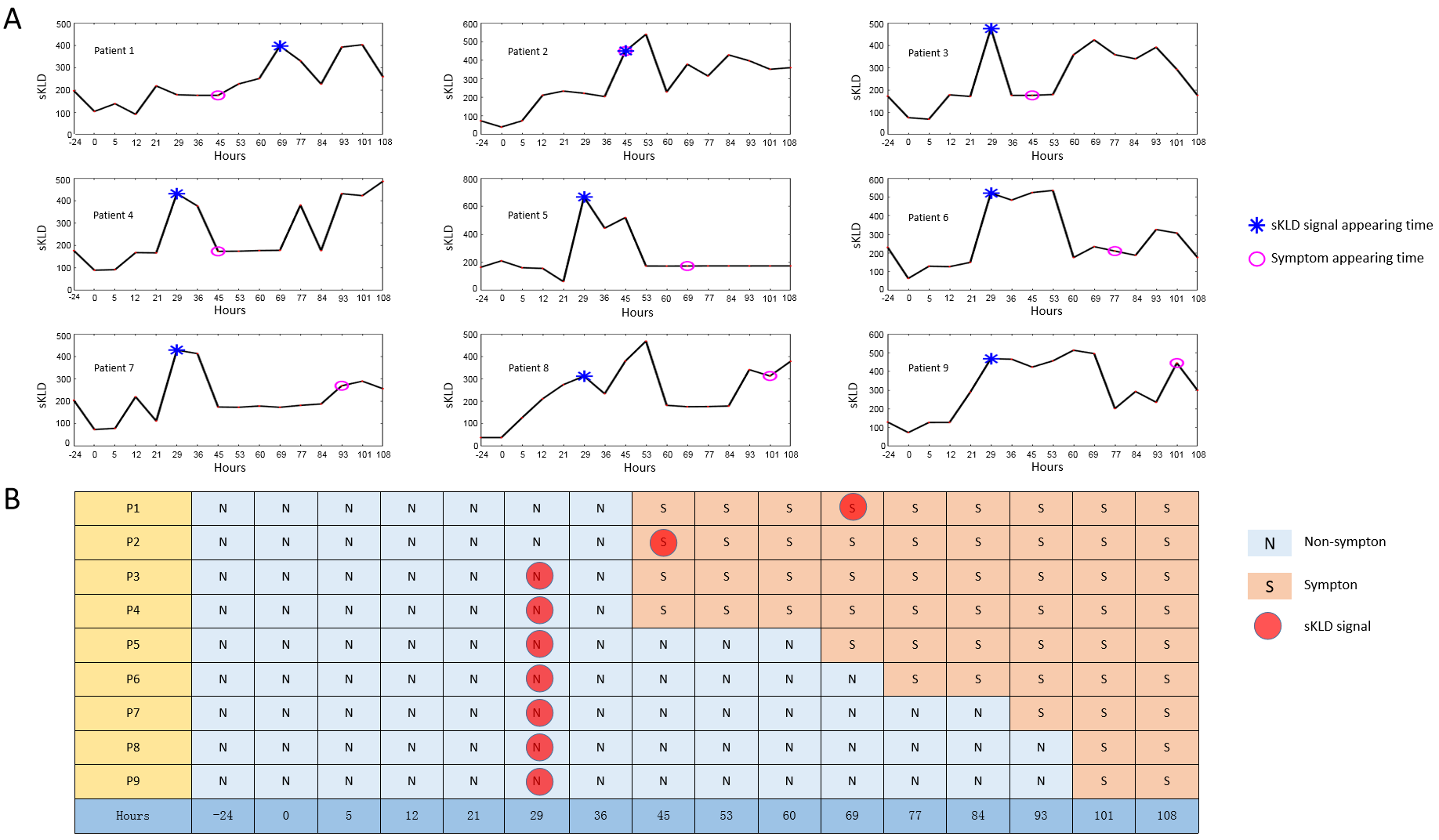


Figure S7: The application of sKLD in influenza infection dataset. (A) The individual-specific sKLD scores for 9 symptomatic subjects. For each sKLD curve, the blue star represents the time point when sKLD signal arises, while the purple circle represents the time point at which the initial flu symptoms appears. (B) The information of 9 symptomatic subjects in the influenza-infection challenge.

**References**

1. Chen, L., Wang, R., Li, C. & Aihara, K. Modeling Biomolecular Networks in Cells: Structures and Dynamics, (Springer, New York, 2010).

2. Chen, L., Wang, R. & Zhang, X. Biomolecular Networks: Methods and Applications in Systems Biology, (John Wiley & Sons, Hoboken, New Jersey,2009).

3. Becskei, A. & Serrano, L. Engineering stability in gene networks by autoregulation, Nature 405, 590–593(2000).

1. Chen, L. & Aihara, K. Stability of genetic regulatory networks with time delay, IEEE Trans. Circuits Syst. I 49, 602–608(2002).
2. Li, C., Chen, L. & Aihara, K. Stability of genetic networks with SUM regulatory logic: Lur’e system and LMI approach, IEEE Trans. Circuits Syst. I 53,2451–2458(2006).
3. Kloeden, P. & Platen, E. Numerical Solution of Stochastic Differential Equations, (Springer, 1999)
4. O Donovan N, Fischer A, Abdo E M, Simon F, Peter H J, Gerber, H, et al. Differential expression of IgG Fc binding protein (FcgammaBP) in human normal thyroid tissue, thyroid adenomas and thyroid carcinomas. Journal of endocrinology. 2002; 174(3): 517-524. doi: 10.1677/joe.0.1740517
5. Zhao H, Li J, Li X, Han C, Zhang Y, Zheng L, et al. Silencing GPX3 expression promotes tumor metastasis in human thyroid cancer. Current Protein and Peptide Science. 2015; 16(4): 316-321. doi: 10.2174/138920371604150429154840
6. Jin Y, Jin W, Zheng Z, Chen E, Wang Q, Wang Y, et al. GABRB2 plays an important role in the lymph node metastasis of papillary thyroid cancer. Biochemical and biophysical research communications. 2017; 492(3): 323-330. doi: 10.1016/j.bbrc.2017.08.114
7. Liu L, Wang J, Li X, Ma J, Shi C, Zhu H, et al. MiR-204-5p suppresses cell proliferation by inhibiting IGFBP5 in papillary thyroid carcinoma. Biochemical and biophysical research communications. 2015; 457(4): 621-626. doi: 10.1016/j.bbrc.2015.01.037
8. Anania M C, Sensi M, Radaelli E, Miranda C, Vizioli M G, Pagliardini S, et al. TIMP3 regulates migration, invasion and in vivo tumorigenicity of thyroid tumor cells. Oncogene. 2011; 30(27): 3011-3023. doi: 10.1038/onc.2011.18
9. Ye D, Jiang Y, Sun Y, Li Y, Cai Y, Wang Q, et al. METTL7B promotes migration and invasion in thyroid cancer through epithelial-mesenchymal transition. Journal of molecular endocrinology. 2019; 63(1): 51-61. doi: 10.1530/JME-18-0261
10. Li J H, Zhang S Q, Qiu X G, Zhang S J, Zheng S H, Zhang D H. Long non-coding RNA NEAT1 promotes malignant progression of thyroid carcinoma by regulating miRNA-214. International journal of oncology. 2017; 50(2): 708-716. doi: 10.3892/etm.2018.6493
11. Lindquist D, Alsina F C, Herdenberg C, Larsson C, Höppener J, Wang N, et al. LRIG1 negatively regulates RET mutants and is downregulated in thyroid cancer. International journal of oncology. 2018; 52(4), 1189-1197.
12. Shang Y, Chai N, Gu Y, Ding L, Yang Y, Zhou J, et al. Systematic immunohistochemical analysis of the expression of CD46, CD55, and CD59 in colon cancer. Archives of Pathology and Laboratory Medicine. 2014; 138(7): 910-919. doi: 10.5858/arpa.2013-0064-OA
13. Li Z, Lin S, Jiang T, Wang J, Lu H, Tang H,et al. Overexpression of eIF3e is correlated with colon tumor development and poor prognosis. International journal of clinical and experimental pathology. 2014; 7(10): 6462 - 6474.
14. Baik I H, Jo G H, Seo D, Ko M J, Cho C H, Lee M G, et al. Knockdown of RPL9 expression inhibits colorectal carcinoma growth via the inactivation of Id-1/NF-κB signaling axis. International journal of oncology. 2016; 49(5): 1953-1962. doi: 10.3892/ijo.2016.3688
15. Moriwaka Y, Luo Y, Ohmori H, Fujii K, Tatsumoto N, Sasahira T, et al. HMGB1 attenuates anti-metastatic defense of the lymph nodes in colorectal cancer. Pathobiology. 2012; 77(1), 17-23. doi: 10.1159/000272950
16. Tang J, Zhang L, She X, Zhou G, Yu F, Xiang J, et al. Inhibiting CD164 expression in colon cancer cell line HCT116 leads to reduced cancer cell proliferation, mobility, and metastasis in vitro and in vivo. Cancer investigation. 2012; 30(5): 380-389. doi: 10.3109/07357907.2012.666692
17. Lugli A, Iezzi G, Hostettler I, Muraro M G, Mele V, Tornillo L, et al. Prognostic impact of the expression of putative cancer stem cell markers CD133, CD166, CD44s, EpCAM, and ALDH1 in colorectal cancer. British journal of cancer. 2010; 103(3): 382 - 390. doi: 10.1038/sj.bjc.6605762
18. Laudato S, Patil N, Abba M L, Leupold J H, Benner A, Gaiser T, et al. P53‐induced miR‐30e‐5p inhibits colorectal cancer invasion and metastasis by targeting ITGA6 and ITGB1. International journal of cancer. 2017; 141(9): 1879-1890. doi: 10.1002/ijc.30854
19. Duan L, Wu R, Zou Z, Wang H, Ye L, Li H, et al. S100A6 stimulates proliferation and migration of colorectal carcinoma cells through activation of the MAPK pathways. International journal of oncology. 2014; 44(3): 781-790. doi: 10.3892/ijo.2013.2231

23. Yu, X., Zhang, J., Sun, S., et al. Individual-specific edge-network analysis for disease prediction. Nucleic acids research. 2017; 45(20), e170-e170.
